# An overview of the role of radionuclides in targeted cancer treatment: application of biomarkers for patient selection and developments to improve treatment efficacy

**DOI:** 10.3389/fonc.2025.1572118

**Published:** 2025-09-19

**Authors:** Tim A. D. Smith

**Affiliations:** ^1^ Division of Cancer Sciences, University of Manchester, Manchester, United Kingdom; ^2^ Nuclear Futures Institute, School of Computer Science and Engineering, Bangor University, Bangor, United Kingdom

**Keywords:** targeted radionuclide therapy, radionuclide, antibody, cancer, PSMA, biomarker, radiosensitiser

## Abstract

Radiopharmaceuticals for targeted radionuclide therapy (TRT) of tumours consist of a radionuclide conjugated to a component that can target the cancer. Several TRT radiopharmaceuticals have been licensed for the treatment of lymphoma, neuroendocrine and prostate cancers. The outcomes from two TRT trials, NETTER for neuroendocrine and VISION for prostate cancer, demonstrated beneficial outcomes. These findings have increased interest in the application of TRT in the treatment of prostate cancer and expansion to other cancer types. Patient selection for TRT is based on a measure of the overexpression of a target receptor on the cancer. To facilitate this, imaging is carried out using a similar targeting moiety to that used for treatment but labelled with an imaging radionuclide. Theragnostic pairs are selected to enable imaging and treatment with the same construct providing accurate predictions of the pharmacokinetics of the therapeutic in patients. This review covers the imaging biomarkers that act as companion diagnostics for TRT pharmaceuticals and the development of radiopharmaceuticals targeting other cancer types enabling expansion of TRT to these cancers. These include strategies to target cancer cells specifically and a pan-cancer approach by targeting fibroblast-activated protein (FAP) upregulated on cancer-associated fibroblasts (CAF). FAP-targeted radiopharmaceuticals are useful for diagnosis and staging but have drawbacks for TRT. Approaches to improve the efficacy of TRT including the use of high linear energy transfer (LET) alpha-emitters and pre-targeting and combination treatments are also covered. As described in this review, not all patients benefit from TRT making the case for predictive biomarkers. This is particularly important for the more damaging alpha emitters.

## Introduction

Treating cancers using radionuclides began in the 1940s with the use of radioiodine to treat hyperthyroidism and thyroid cancer, exploiting the affinity of thyroid tissue for iodine, a property maintained by some thyroid cancers (1). The β-emitter phosphorus-32 (^32^P) has been used in the form of [^32^P]H_3_PO_4_ for several decades to treat some blood disorders, including polycythaemia vera and bone pain from metastatic cancer (2). More recently, in 2013, [^223^Ra]RaCl_2_, commercially known as Xofigo^®^, was approved by the Food and Drug Administration (FDA) to treat bony metastasis in patients with advanced prostate cancer (3). Each of these applications uses the diseased tissue’s elemental affinity to concentrate radioactive versions of the element or their mimetics.

**Figure 1:**
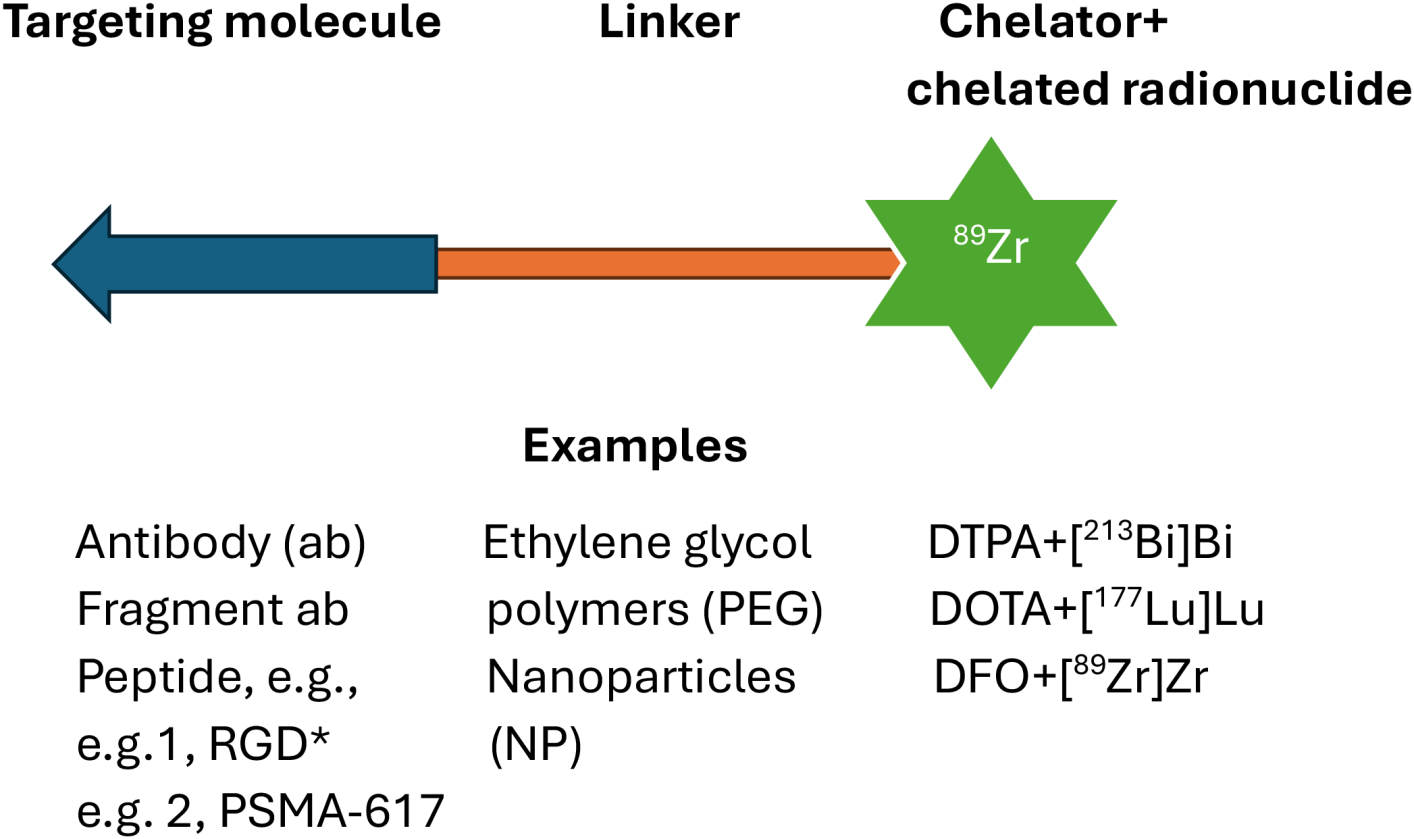
Diagrammatic representation of a targeted radionuclide therapy (TRT) radiopharmaceutical radiolabelled with metal radionuclides. Targeting groups include antibodies, truncated antibodies, and peptides. Examples of linkers include short linear molecules, polyethylene glycol (as monomer or polymer), and a nanoparticle. Metal radionuclides (as in the example in the diagram) are captured in a chelator conjugated to the linker. *Arginylglycylaspartic acid.

Many radiopharmaceuticals consist of complex constructs composed of a targeting moiety attached to a radionuclide via a carrier (see **Figure 1**). The structure of the carrier depends on the radionuclide. Medical radionuclides are often a metal and require chelation. Commonly used chelators include diethylenetriamine pentaacetic acid (DTPA) for technetium-99m (^99m^Tc) and bismuth-213 (^213^Bi) (4); deferoxamine (DFO) for zirconium-89 (^89^Zr); macrocyclic chelators including DOTA for the trivalent radiometals such as gallium-68 (^68^Ga), scandium-44 (^44^Sc), yttrium-90 (^90^Y), lutetium-177 (^177^Lu), and other radio-lanthanides including actinium-225 (^225^Ac); and 1,4,7-triazacyclononane-1,4,7-triacetic acid (NOTA) for Ga and copper (Cu) radionuclides (5).

The targeting moiety can be a peptide, an antibody (Ab), or smaller-sized engineered antibody mimetics. These small constructs include fragment antibody (scFv) and minibodies (Mb) (two scFv to increase avidity), and affibodies—small robust protein binders and nucleic acid-based structures called aptamers (6–8). Affibodies are approximately 7 kDa in size and have picomole (pM) target affinity (9). However, they can demonstrate high kidney uptake due to reabsorption by the renal tubules (9). Modifications to the amino acid sequence, hydrophilicity, surface charge, chelate, and radionuclide can improve tumour/non-tumour uptake ratios (9).

For this review, papers from the past 5 years (mostly) were selected from a “Web-of-Science” search using the following keywords: targeted radiotherapy, molecular radiotherapy, radioimmunotherapy, and cancer. The purpose of this review is to provide a background for non-specialists in these areas and a comprehensive survey of the current research and clinical state of targeted radionuclide therapy (TRT).

## Radionuclides commonly used in medicine

**Table 1** shows examples of radionuclides commonly used in nuclear medicine. Imaging radionuclides are γ-emitters for single-photon emission computer tomography (SPECT) or positron (β^+^) emitters for positron emission tomography (PET). Technetium-99m (^99m^Tc) is used in the majority (>80%) of nuclear medicine scans due to its practical half-life (t_1/2_ = 6 h) and ideal imaging γ-emission energy of 140 keV, with high detection efficiency (10). Fluorine-18 (^18^F) is the most commonly used PET isotope which has a low positron energy (0.6 MeV) and produces high-resolution images (11). The positron-emission tomography (PET) radionuclides with short t_1/2_s, fluorine-18 (^18^F) and gallium-68 (^68^Ga) (t_1/2_ of 110 min and 68 min, respectively), are suitable for labelling small ligands such as peptides, which demonstrate rapid (hours) blood clearance. Antibodies with longer blood residence times (days) are labelled with longer-lived radionuclides, particularly Zirconium-89 (^89^Zr) with a t_1/2_ of 78 h (12).

**Table 1:** Examples of radionuclides used clinically with characteristics.

Emission	Range	LET	Examples (range in tissue - for β-emitters)	Use
γ			^111^In, ^99m^Tc	Imaging
Positron (β^+^)			^89^Zr, ^18^F	Imaging
β	Up to 10 mm	low	^177^Lu (1mm), ^90^Y (10 mm)	TRT
α	Up to 100 μm	high	^223^Ra, ^225^Ac	TRT
Auger electrons	<0.5 µm	high	^125^I, ^89^Zr, ^111^In, ^123^I	TRT

Zr, zirconium; In, indium; Tc, technetium; Lu, lutetium; Y, yttrium; Ra, radium; Ac, actinium; I, iodine.

Patients selected for TRT have pretreatment SPECT or PET scans to ensure tumour expression of the target receptor. Therapy with radionuclides is delivered using β- or α- and less commonly Auger emitters (13). The deposition of energy within the tumour from radioactive decay causes DNA damage. When unrepaired or incorrectly repaired, DNA damage can result in cell death. High linear energy transfer (LET), characteristic of α- and Auger emissions, results in high-density localised DNA damage (13), whereas sparsely ionising but longer range β-emitters improve radiation dose heterogeneity across tumours (14).

## Production of radionuclides

Most medical radioisotopes are produced in a nuclear reactor (e.g., ^177^Lu), or through proton or ion particle bombardment in a cyclotron (15, 16). The imaging radionuclide ^99m^Tc is produced in generators from molybdenum-99 [^99^Mo], delivered weekly to nuclear medicine departments (17). There is currently great interest in treating patients with neuroendocrine tumours (NENs) and other cancers using Actinium-225 (^225^Ac)-labelled radiopharmaceuticals (18). Actinium-225 is obtained as a decay product of ^229^Th of which there is a limited supply (enough to produce 68 GBq/year of ^225^Ac—just a few hundred patient doses) (19). Several production pathways attempting to overcome supply limitations of ^229^Th include neutron irradiation of ^226^Ra and accelerator routes (19). **Table 2** shows fabrication routes for other clinically used radionuclides and ones with potential clinical application. Isotopes of terbium have great potential for imaging and therapy, but their production is problematic. The reader is referred to a comprehensive review on production routes for Tb radioisotopes (47).

**Table 2:** Established and novel methods for production of radionuclides.

Radionuclide (emission)	Reaction	Cyclotron/neutrons/ Generator	Reference
Diagnostic radionuclides			
Positron emitters			
^64^Cu (β^+^, β^−^ t_1/2_ = 12.7 h)	^63^Ni (p, γ) ^64^Cu ^64^Ni (p, n) ^64^Cu* ^65^Cu (p, pn) ^64^Cu ^67^Zn (p, α) ^64^Cu	Medium flux nuclear reactor Cyclotron Cyclotron 18 MeV Cyclotron 18 MeV	(20) (21) (21) (21)
^48^V (β^+^ t_1/2_ = 16 d)	^Nat^Ti (p, n) ^48^V	Cyclotron 18 MeV	(22)
^43^Sc (β^+^, EC t_1/2_ = 3.9 h)	^42^Ca (d, n) ^43^Sc ^Nat^Ca (α, p) ^43^Sc	Cyclotron 18 MeV	(23) (24)
^44^Sc (β^+^ E_ave_ 0.63 MeV t_1/2_ = 3.93 h)	^44^Ca (p, n) ^44^Sc	Cyclotron (15–25 MeV)	(25)
^72^As (β+ 2.47 MeV t_1/2_ = 26 h)	^72^Ge (p, n) ^72^As	Cyclotron	(26)
^66^Ga (β^+^ t_1/2_ = 9.49 h)	^66^Zn (p, n) ^66^Ga	Cyclotron	(27)
^69^Ge (β^+^, β^−^ E_max_ = 1.2 MeV t_1/2_ = 39 h)	^69^Ga (p, n) ^69^Ge	Cyclotron	(28)
^124^I (γ, β^+^, t_1/2_ = 4.2 h)	^124^Te (p, n) ^124^I	Cyclotron	(29)
^89^Zr (β^+^, t_1/2_ = 3.3 d)	^89^Y (p, n) ^89^Zr	Cyclotron	(30)
SPECT radionuclides			
^123^I (EC γ, t_1/2_ = 13.3 h)	^127^I (p, 5n) ^123^Xe, ^123^Xe decays to ^123^I	Cyclotron	(31)
^111^In (EC γ, t_1/2_ = 2.8 d)	^112^Cd (p, 2n) ^111^In	Cyclotron	(32)
^99m^Tc (EC γ, t_1/2_ = 6 h)	Decay of ^99^Mo	Generator	(17)
Therapeutic radionuclides			
^90^Y (β^−^, t_1/2_ = 64 h)	^90^Sr (β^−^) ^90^Y	Decay of ^90^Sr	(33)
^177^Lu (β^−^, γ, t_1/2_ = 6.65 d)	^176^Lu (n, γ) ^177^Lu	Nuclear reactor	(34)
^223^Ra (α, t_1/2_ = 11.4 d)	^227^Ac (α, β) ^223^Ra	Generator	(35)
^213^Bi (α, t_1/2_ = 46 min)		Generator; parent ^225^Ac	(36)
^225^Ac (α, t_1/2_ = 9.92 d)	Purified from ^229^Th ^226^Ra (p, 2n) ^225^Ac	Cyclotron 16 MeV	(19)
^211^At (α, EC t_1/2_ = 7.2 h)	^209^Bi (α, 2n) ^211^At	Cyclotron K150	(37)
^32^P (β^−^, t_1/2_ = 14.3 d) E_max_1.7 MeV	^32^S (n, p) ^32^P	Neutron irradiation	(38)
^153^Sm (β^−^ E_max_=0.81MeV γ 103 keV t_1/2_ = 1.93 d)	^152^Sm (n, γ) ^153^Sm	Nuclear reactor	(39)
^67^Cu (β^−^, t_1/2_ = 61.8 h)	^70^Zn (p, α) ^67^Cu	Cyclotron 30 MeV	(40)
^47^Sc (β^−^ E 0.6 MeV t_1/2_ = 3.35 d)	^46^Ca (n, γ)^47^Ca, ^47^Sc ^47^Ti (n, p)^47^Sc ^Nat^V (n, p)^47^Sc	Thermal neutrons High energy neutrons Cyclotron 20 MeV	(41) (41) (42)
^170^Tm (β^−^ E_max_= 0.97 MeV t_1/2_ = 128 d)	^169^Tm (n, γ) ^170^Tm	Thermal neutron flux	(43)
^165^Er (Auger t_1/2_ = 10.4 h)	^165^Ho (p, n) ^165^Er	Cyclotron 16 MeV	(44)
^167^Tm (Auger, γ208 KeV t_1/2_ = 9.25 d)	^168^Er (p, 2n) ^167^Tm	Cyclotron 23 MeV	(45)
^195m^Pt (Auger t_1/2_ = 4 d)	^195^Pt	Neutron activation	(46)

^1^Enriched ^64^Ni is expensive.

Bi, bismuth; S, sulphur; P, phosphorus; Er, erbium; Ho, holmium; Ni, nickel; Cu, copper; Zn, zinc; Ga, gallium; Ge, germanium; At, astatine; V, vanadium; Ti, titanium; Sc, scandium; Ca, calcium; Ac, actinium; Sm, samarium; Tm, thulium; Pt, platinum; At, astatine; Te, tellurium.

## Theragnostic systems: imaging and therapy with the same construct

Imaging biomarkers include measures of the uptake of single-photon emission computer tomography (SPECT) and positron emission tomography (PET) tracers based on diagnostic analogues of TRT therapeutics (48). The tissue/tumour uptake is usually defined as a standardised uptake value which is uptake per gram tissue per injected dose of the tracer. Tracers based on TRT molecules are used as part of a TRT inclusion criteria and for dosimetry prediction and dose tailoring of the TRT (48).

**Figure 2:**
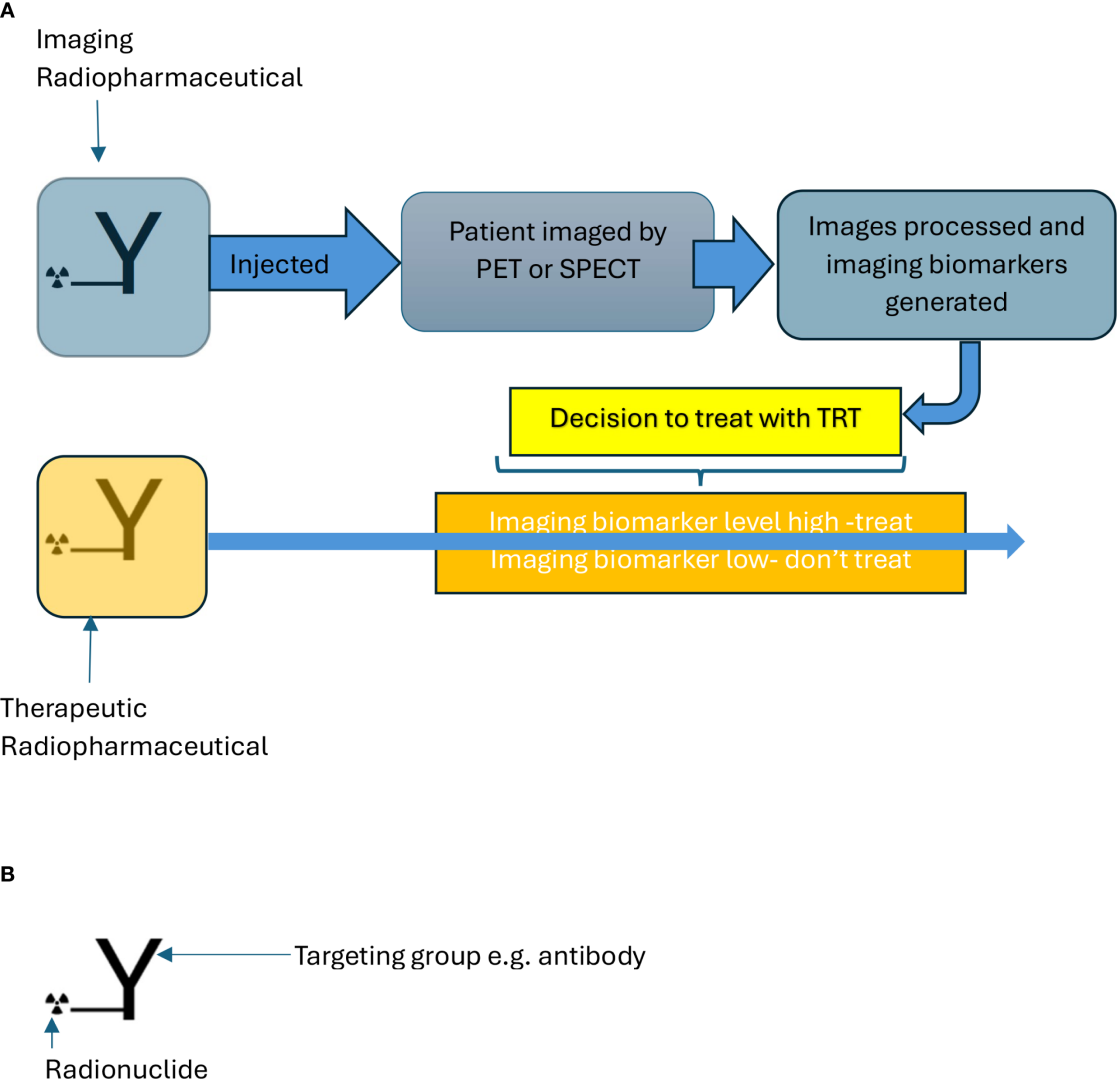
**(A)** Scheme showing decision to treat with TRT (targeted radionuclide therapy) based on imaging. **(B)** Radiopharmaceutical targets cancer cells with radionuclide for imaging and therapy. The molecule for imaging and therapy is identical for a theragnostic except for the radionuclide.

Theragnostic strategies involve imaging and treatment of the tumour using the same radiopharmaceutical (if the radionuclide has an imaging emission) or imaging with an otherwise identical radiopharmaceutical as for TRT but labelled with an imaging radioisotope (theragnostic pair) (49). Tumour uptake and biodistribution of the imaging radiopharmaceutical informs on the potential benefit of TRT for that patient (see **Figure 2**).

Theragnostic systems are a rapidly growing component of nuclear medicine combining radionuclide interchangeability to facilitate imaging and therapeutic capability (49). In addition to confirming patient suitability for TRT, pretreatment imaging allows predictive dosimetry to tailor administered therapeutic dose (50). Radioiodine, which has a long history in nuclear medicine has a multitude of isotopes. The commonly used radioiodine nuclides for medical applications, ^123^I, ^124^I, ^125^I, and ^131^I, between them achieve single-photon emission computed tomography (SPECT) imaging, positron emission tomography (PET) imaging from gamma, positron (β^+^) decay properties, Auger, and β-emission for targeted radiotherapy (51).

Many radionuclides are metals and require chelation to enable stable integration into a radiopharmaceutical. Some metals, for example, scandium (Sc), have multiple isotopes suitable for imaging and treatment (52). Thus, ^43^Sc (t_1/2_ = 3.9 h) and ^44^Sc (t_1/2_ = 4 h) are positron emitters whereas ^47^Sc (t_1/2_ = 3.35 d) is a β-emitter, suitable for therapy and a primary gamma-emission suitable for SPECT. Scandium forms stable complexes with the chelator, 1,4,7,10-tetraazacyclododecane-1,4,7,10-tetraacetic acid (DOTA), but complexation requires heating to 80°C (52). Terbium has four particularly valuable radionuclides for nuclear medicine ^149^Tb (t_1/2_ = 4.2 h, α, β^+^-emitter), ^152^Tb (t_1/2_ = 17.5 h, EC and β^+^ emitter), ^155^Tb (t_1/2_ = 5.3 d, EC SPECT), and ^161^Tb (t_1/2_ = 6.9 d, β^−^, Auger) (47).

Chelators which allow coordination of chemically diverse metals are particularly useful, allowing complexation of radiometals with different emission characteristics for broad-range theragnostic systems. Simms et al. (53) have recently developed a chelator that can bind to [^225^Ac]Ac^3+^, [^177^Lu]Lu^3+^, [^111^In]In^3+^, or [^44^Sc]Sc^3+^, enabling delivery of treatment with α- and β-emitters and imaging with SPECT and PET, respectively, on the same molecule.

Imaging ^223^Ra-SPECT generates poor images due to the small photon abundance and scarcity of the injected activity (54). Due to the similar co-ordination chemistry of Ra and Ba, it has been suggested that ^223^Ra could form a theragnostic pair with ^131^Ba (t_1/2_ = 11.5 d) and ^135m^Ba (t_1/2_ = 28.7 d). ^131^Ba and ^135m^Ba decay by electron capture with γ-emissions of 124 keV (30%) and 268 keV (16%), respectively. Suitable multidendate chelators are being produced (54). An alternative approach to chelation includes encapsulation of [^223^Ra] by hydroxyapatite nanoparticles (55) and/or linkage to nanoparticles (56).

The chelator DOTA can form stable chelates with cerium (Ce), Th, and Ac (57). Cerium-134 decays (t_1/2_ = 3.2 days) to the positron emitter ^134^La (t_1/2_ = 6 min). The *in vivo* generator ^134^Ce/^134^La enables application of the short-lived ^134^La as a PET imaging nuclide for both alpha-emitting ^225^Ac and ^227^Th radionuclides. MicroPET images of [^134^Ce]Ce-DOTA-Trastuzumab demonstrated high tumour uptake and low bone and liver uptake analogous to previously reported [^225^Ac]Ac-DOTA-Trastuzumab biodistribution results (57).

## Preclinical/early clinical TRT radiopharmaceuticals

Experimental approaches to treating prostate cancer with TRT, explored at the preclinical stage, include using different prostate-specific membrane antigen (PSMA) ligands, radionuclides, or targeting other receptors upregulated on prostate cancer cells (23, 58–60). Garnuszek et al. (58) radiolabelled PSMA-D4 with the three β-emitters ^177^Lu, ^90^Y, ^47^Sc, and the α-emitter ^225^Ac. All radiocomplexes demonstrated high accumulation in LNCaP tumour xenografts and rapid clearance from blood and non-target tissues. Scandium-47 forms a theragnostic pair with the β^+^ -emitting ^43^Sc. ^43^Sc- and ^47^Sc-PSMA-617 demonstrate congruous uptake by LNCaP-ENZaR xenografts (23). Other α-emitting radionuclides including, ^212^Pb and thorium-227 (^227^Th) (t_1/2_ = 18.7 days), and the radiohalogen, astatine-211 (^211^At), have been investigated in preclinical research targeting PSMA (59). Böhnke et al. (60) fabricated a PSMA ligand from PSMA-617 with a modified linker and chelator (carboxy-HOPO), which demonstrated stable chelation of ^227^Th, high uptake by PSMA-expressing tumours in mice, and fast renal clearance.

Prostate cancer overexpresses other cell surface proteins besides PSMA. These include the proteases human kallikrein peptidases (HK2 and HK3) and receptors such as delta-like ligand 3 (DLL-3), CD46, and CUB domain-containing protein 1 (CDCP1) (59). Prostate stem cell antigen (PSCA) which is overexpressed on metastatic prostate, pancreatic, and bladder cancer cells is an alternative target to PSMA (61). Antibodies conjugated to chelated metals are internalised on binding to surface receptors on both tumour and non-tumour cells and the metal retained in the lysosomal compartment. Tsai et al. (7) compared the anticancer efficacy and biodistribution of the anti-PSCA minibody, A11 Mb labelled by iodination with ^131^I, which is not retained, with a [^177^Lu]-DTPA. Both exhibited similar cancer cell killing *in vivo*. However, dosimetry, determined using immunoPET studies with ^124^I and ^89^Zr as surrogates for ^131^I and ^177^Lu, demonstrated clearance of ^124^I from liver and kidneys but retention of ^89^Zr by kidneys. This suggests that ^131^I is a better choice for delivering tumour-inhibitory radiation dose but minimising non-tumour retention (7). However, trans-iodination associated with halogen-labelled radiopharmaceuticals can result in non-tumour exposure.

Studies of peptides targeting the gastrin-releasing peptide receptor (GRPR), on the surface of localised and metastatic prostate cancers, are also underway (62). [^64^Cu]Cu-SAR-BBN is in clinical development for PET imaging of GRPR-expressing prostate cancer using bombesin peptides. A preclinical study has shown that Cu-SAR-BBN labelled with the therapeutic radionuclide [^67^Cu] is an effective treatment for PC-3 tumours (62). Radiolabeled GRPR antagonists are considered safer than agonists and have shown higher tumour uptake and clearance than agonists. Kanellopoulos et al. (63) labelled GRPR antagonists with ^99m^Tc radiotracers demonstrating high uptake by GRPR-expressing tumours.

Rapid clearance of small-molecule ligands such as [^225^Ac]Ac-PSMA-617 can limit tumour delivery (64). To increase circulatory residence time, albumin-binding entities can be incorporated into radiopharmaceuticals enabling binding to circulatory albumin. The benefit of this approach was examined in [^225^Ac]Ac-PSMA-ligands ([^225^Ac]Ac-SibuDAB) (64) and [^64^Cu]Cu-PSMA-BCH (65). Both agents demonstrated increased circulation time and tumour uptake (64, 65). Response of xenografts bearing PSMA-expressing cancer cells was greater in mice injected with [^225^Ac]Ac-SibuDAB compared with [^225^Ac]Ac-PSMA-617 (64). Uptake by normal tissue was also greater but did not result in greater toxicity (64).

Dosimetry modelling can be highly informative and contribute to decisions regarding radionuclide of choice. A modelling study of the α-emitting ^225^Ac, ^211^At, ^212^Pb, ^223^Ra, and ^227^Th and the β-emitting therapeutic radionuclides ^67^Cu, ^131^I, ^177^Lu, and ^90^Y demonstrated that the β- and α-emitters ^177^Lu and ^211^At respectively are most suited for prostate radionuclide therapy because they can reduce toxicity exposure to surrounding organs but provide sufficient dose to treat the prostate tumour (66).

**Table 3** summarises many of the different proteins upregulated on other cancer types and the imaging and TRT radiopharmaceuticals for their targeting that are in preclinical and in some cases clinical trials.

**Table 3:** Targets preclinically evaluated as TRT for different cancers.

Target	Cancer type	Ligand	Radionuclide	Reference
PD-1/PD-L1 (programmed death receptors)	Non-small cell lung carcinoma; melanoma; colorectal cancer (CRC)	PD-1	[^68^Ga]	(67)
Neurotensin receptor	CRC	Neurotensin	[^68^Ga], [^177^Lu]	(68)
Gonadotrophin-releasing factor receptor (GRFR)	GRFR-expressing cells, e.g., prostate, breast, ovarian	Leuprolide	[^68^Ga], [^177^Lu]	(69)
Insulin-like growth factor-1 (IGF1) receptor	Several cancer types	IGF1 receptor affibodies	[^68^Ga], [^111^In]	(70)
IGF2R receptor	Osteosarcoma	IGFR2-Fab^1^	[^225^Ac], [^111^In]	(71)
Glypican-3 (GPC3)	Hepatocellular carcinoma (HCC)	RAYZ8009 (macrocyclic peptide)	[^177^Lu], [^225^Ac]	(72)
	Hepatocellular carcinoma (HCC)	Anti-GPC3-Ab (αGPC3)	[^227^Th]	(73)
	Glioblastoma	GC33^2^ Mab vs. scvf^3^	[225Ac] [89Zr]	(74)
Nectrin-1	Breast cancer	NP137 Mab	[^111^In], [^177^Lu]	(75)
CXCR4	Non-small cell lung carcinoma (NSCLC)	AMD3465	[^99m^Tc]	(76)
Brevican (matrix protein) isoform dg-Bcan	Glioblastoma	BTP7	[^18^F]	(77)
Mutant K-Ras cells: K-Ras4B oncogenic isoform bind PDE-6	Colorectal cancer (CRC)	Inh of K-Ras4B/PDE-6 dissociation (I-C19)	[^131^I]	(78)
DLL3 Notch inhibitory ligand	Small cell lung carcinoma (SCLC)	Anti-DLL3 antibody SC16	[^177^Lu]	(79)
Carbonic anhydrase (CA9)^4^	CRC	Dual: CA9 inhibitor acetazolamide+CA9 probe	[^111^In]	(80)
	Renal cell carcinoma	CA9 Ab	[^111^In]	(81)
PDGFRβ	Carcinomas	Affibodies	[^68^Ga]	(82)
VPAC1 receptor	Epithelial cancers	VP2 small fusion protein binds VPAC1	[^211^At]^5^	(83)
	Bladder cancer	TP3805	[^64^Cu]	(84)
Tissue factor (TF)	Gastric cancers	Anti-TF Ab	[^211^At]	(85)
CD123	Acute leukaemia	Anti-CD123 Abs	[^211^At]	(86)
CD13+angiogenesis related amino-peptidase (APN)	Angiogenic tumour vessels	Dual: asparagine-glycine-arginine (NGR) +APD	[^68^Ga]	(87)
Death receptor 5 (DR5)	Colorectal cancer	Anti-DR5 Ab (CTB006)	[^89^Zr], [^177^Lu]	(88)
CDCP1	CRC	Anti-CDCP1 Ab	[^89^Zr]	(89)
Gastrin-releasing peptide receptor (GRPR)	Gastrointestinal stromal tumour (GIST)	NeoB–GRPR binding protein	[^177^Lu]	(90)

^1^Fab, fragment antibody. ^2^Modest survival benefit for mice bearing HepG2 tumours but marked haematological toxicity due to long circulatory residence time of antibodies. ^3^Both internalised but greater uptake with whole Ab. ^4^Carbonic anhydrase is a cellular biomarker of hypoxia. ^5^High stomach uptake—late toxicity in mice.

Most targeted treatments are tailored to cancer type, but an approach that could lead to a pan-cancer TRT radiopharmaceutical exploits targeting the non-tumour cells present in the tumour microenvironment, particularly cancer-associated fibroblasts which overexpress the fibroblast-activating protein.

The tumour microenvironment, in addition to cancer cells, consists of immune cells, cancer-activated fibroblasts (CAFs), and host epithelial cells (91). Overwhelming evidence indicates that CAFs, or at least some CAF subtypes, are tumour promoting (92). Fibroblast activation protein (FAP) is a type II membrane-bound glycoprotein overexpressed in CAFs and is highly expressed in the stromal compartments of several malignant cancers (92).

The tumour inhibitory potential of FAP inhibitors has been explored but proved not particularly effective. However, FAP inhibitors radiolabelled with ^68^Ga, e.g., [^68^Ga]Ga-FAPI-46, are informative for the diagnosis and staging of several cancer types (93–97). Oster et al. (93) demonstrated in patients with glioblastomas a correlation between histological FAP expression and tumour SUV mean and SUV peak of [^68^Ga]Ga-FAPI-46 using PET. Higher FAP expression was present in a gliosarcoma subgroup, suggesting that [^68^Ga]Ga-FAPI-46 uptake may be useful diagnostically. Unterrainer et al. (94) demonstrated that [^68^Ga]Ga-FAPI-46-PET could be beneficial in lymph node staging for patients with bladder cancer. Compared with CT alone, [^68^Ga]Ga-FAPI-46-PET/CT improved the accuracy of staging resulting in major changes in the treatment of patients with oesophageal cancer (95) and pancreatic cancer (96). [^68^Ga]Ga-FAPI-46-PET scans highlighted metastasis that were not evident in FDG-PET scans. However, for patients with NENs or prostate cancers, imaging of patients with [^68^Ga]Ga-FAPI-PET is less informative than [^68^Ga]Ga-DOTATATE (98) or [^68^Ga]Ga-/[^18^F]F-PSMA-11 (99), respectively.

Recent studies have explored radiolabelled FAP inhibitors or antibodies to FAP for tumour treatment. Kuyumcu et al. (100) demonstrated that the mean absorbed dose to organs at risk with the ^177^Lu-labelled quinoline-based inhibitor of FAP, FAPI-04, in four patients with metastatic cancers is reasonably low. FAPI-46, which has longer tumour residence times than FAPI-04 (101), radiolabelled with ^90^Y (102–104) and ^177^Lu (105), have been recently explored in dose-escalation studies with limited patient numbers. Nine patients with high FAP-expressing metastatic soft-tissue or bone sarcoma or pancreatic cancers who had exhausted other conventional treatments were given [^90^Y]Y-FAPI-46 (102). Three patients received one treatment cycle, and six received two cycles. Radiographic disease control was evident in four patients. At-risk organ doses were low. In a larger study of a cohort of 21 patients with different cancers treated with [^90^Y]Y-FAPI-46, partial response in one patient and stable disease in seven was reported (103). Thrombocytopenia and anaemia were evident in several patients. Patients with solitary fibrous tumours (11) received two or three cycles of [^90^Y]Y-FAPI-46 (104). Disease control was evident in 9 of the 11 patients. A dose-escalation study of [^177^Lu]Lu-FAPI-46 in 18 patients with FAP-expressing inoperable or refractory metastatic cancers (105) demonstrated good tolerance. Patients received 1.4 GBq increasing to 4.4 GBq of [^177^Lu]Lu-FAPI-46 with no toxicity evident in most patients.

Short retention times compromise the therapeutic efficacy of some FAP inhibitors and potentially the efficacy of TRT via FAP. Several strategies to improve retention have been explored preclinically including ligand dimerization, covalent bond formation at the binding site, and the use of antibodies to FAP.

To improve binding, Zhong et al. (106) produced a dimerised version of FAPI-04 and compared tumour uptake of the monomer and dimer labelled with ^68^Ga and ^177^Lu using micro-PET. Images of SKOV3, A431, and H1299 xenografts revealed that tumour uptake of the dimer was greater than by the monomer. They also showed that [^177^Lu]Lu(FAPI-04)(2) effectively reduced tumour growth. Pang et al. (107) compared tumour uptake of dimer and tetramer versions of FAPI-46 labelled with ^68^Ga by FAP-expressing xenografts. They demonstrated far greater uptake and lower washout of the tetramer compared with the dimer. Tumour growth inhibition by [^177^Lu]Lu-FAPI-04) was significantly (p<0.001) greater than by [^177^Lu]Lu-FAPI-46 monomer. The first-in-human study of a [^177^Lu]-labelled FAPI dimer was given to patients with treatment refractory breast, thyroid, or paraganglioma cancers (108). The study demonstrated increased residence time and higher median lesion absorbed dose using a dimer version of FAPi (6.7 Gy/GBq) compared with the monomer (0.6 Gy/GBq).

The FAPI FAP-2286 is a cyclic peptide with high-affinity FAP-binding characteristics. PET/CT scans of 21/21 patients with different malignancies including breast, pancreatic, and thyroid cancer demonstrated uptake of [^68^Ga]Ga-FAP-2286 within primary solid tumours, adjacent excised tissues, and metastatic lesions (109), suggesting the potential of a general cancer tracer. The first-in-human study of [^177^Lu]Lu-FAP-2286 for peptide-targeted radionuclide therapy (PTRT) was carried out on 11 patients with advanced adenocarcinomas of the pancreas, breast, rectum, or ovary with prior confirmation of uptake on [^68^Ga]Ga-FAP-2286 or [^68^Ga]Ga-FAPI-04 PET/CT. [^177^Lu]Lu-FAP-2286 was well tolerated and demonstrated significant retained tumour uptake (110).

Most TRT is mediated through the interaction of a radionuclide conjugated ligand or antibody to an overexpressed cell surface receptor via a temporary non-covalent interaction. To increase the duration of target exposure to the radionuclide, radiopharmaceuticals that interact and form covalent bonds with the target receptor have been examined in preclinical work. Cui et al. (111) modified a FAP inhibitor by inclusion of an aryl fluorosulphate (FS) to covalently link with nucleophilic centres in the binding site of FAP. The FS-modified [^177^Lu]Lu-FAPI demonstrated 2.5× higher tumour uptake, 13× longer retention, and significantly greater tumour control compared with [^177^Lu] Lu-FAPI.

Xu et al. (112) developed a theragnostic pair targeting FAPα with two novel recombinant anti-FAPα antibodies labelled with ^89^Zr and ^177^Lu. PET/CT and SPECT/CT imaging of the ^89^Zr and ^177^Lu antibodies AMS002-1-Fc respectively demonstrated good tumour uptake by both and tumour control by [^177^Lu] Lu-AMS002-1-Fc. Sibrotuzumab is an anti-FAP monoclonal antibody that was trialled for metastatic colorectal cancer but failed to show benefit. However, Xu et al. (112, 113) demonstrated using PET/CT, high uptake, and retention of the ^89^Zr-labelled derivative of sibrotuzumab, PKU525, by FAP-expressing xenografts. They also showed correspondingly high tumour uptake of [^177^Lu]Lu-DOTA-NCS-PKU525, e.g., 23% and 33% ID/g at 24 and 96 h, respectively, and achieved tumour growth inhibition with a single 3.7-MBq dose.

Liu et al. (114) produced a FAPI which could be radiolabelled with short t_1/2_ imaging ^18^F, or therapeutic ^213^Bi to pair the rapid kinetics. The inhibitor included an organotrifluoroborate linker which increased cell internalisation. FAPI has also been radiolabelled with ^211^At (115).

Early clinical trials of radiolabelled FAP ligands for the treatment of FAP-expressing cancers indicate that they are well tolerated. Developments in FAPI molecules that enhance tumour residence time may improve their therapeutic potential. Evidence suggests that prostate cancers and NENs are more effectively targeted, respectively, with PSMA ligands and somatostatin analogues than FAPIs. However, FAP targeting may be a useful way forward for the application of TRT to other cancer types.

## Licensed cancer-targeting TRT radiopharmaceuticals

There are licensed TRT radiopharmaceuticals available to treat three types of malignancy, namely, B-cell lymphoma and neuroendocrine and prostate cancers. B-cell malignancies can be treated with licensed antibody-based TRT radiopharmaceuticals targeting upregulated receptors including CD20, whereas neuroendocrine and prostate cancers can be treated with licensed small-molecule TRT radiopharmaceuticals targeting the somatostatin receptor and PSMA, respectively.

Several anti-CD20 monoclonal antibodies such as rituximab, ofatumumab, or obinutuzumab improved the therapy of B-cell malignancies through mechanisms including cellular cytotoxicity and induction of apoptosis (116). When radiolabelled, these antibodies exploit the radiosensitivity of lymphoma cells with increased benefit (117). The first FDA-approved radiolabelled antibody was the [^90^Y]Y-murine anti-CD20 antibody ibritumomab for the treatment of indolent lymphoma in 2002 (118), followed by [^131^I]I-tositumomab (Bexxar) in 2003 (119). Despite clear patient benefit including long-term remission in many patients and relatively low cost compared with other treatments for lymphoma, the uptake of these treatments is low due to a range of logistical reasons (119).

Most neuroendocrine cancers (NENs) overexpress the somatostatin receptor, which has several subtypes (120). The peptide ligand somatostatin is rapidly degraded in the circulation (121). Degradation-resistant somatostatin analogues including [^111^In]In-DTPA-pentetreotide (Octreoscan^®^) were developed in the 1980s and have been used for several decades for diagnosing neuroendocrine tumours (NENs) (122). However, image interpretation is complicated by high uptake by the liver, spleen, and kidneys. [^111^In]In-DTPA-pentetreotide has been a treatment for NENs due to emission of Auger and internal conversion electrons by ^111^In. This was replaced by β-emitter-labelled somatostatin analogues, e.g., [^177^Lu]Lu-DOTA-TATE, under the name Lutathera^®^, which can also be labelled with ^68^Ga facilitating imaging by PET with improved resolution over SPECT (123). Positive outcomes reported in the NETTER trial for patients with midgut NENs treated with Lutathera^®^ led to approval by the FDA for SSTR2-positive gastroentero-pancreatic NETs (GEP-NETs) in adults (124).

Patients benefit from Lutathera^®^, but many patients will relapse (125). Improving overall treatment outcome may be achieved by several routes. The [^90^Y]-labelled version has been suggested for larger lesions as the β-emissions have a longer range than from [^177^Lu] (126). Approaches using both [^90^Y]- and [^177^Lu]Lu-ligands may be advantageous (127), but combination studies are limited. Improving the radiopharmaceutical using antagonists ([^177^Lu]Lu-DOTA-LM3 and -JR11) (128) which demonstrate greater receptor binding facilitates their application to cancers with lower SSTR density such as breast cancer (129). The use of radionuclides that produce more DNA damage such as the shorter range α-emitters including ^225^Ac may also improve outcomes and decrease normal tissue exposure (130).

Treatment of patients with prostate cancer is dependent on stage (131). Patients with advanced-stage metastatic castration-resistant prostate cancer (CRPC) may receive radionuclide treatments targeting prostate-specific membrane antigen (PSMA) (132). PSMA, a transmembrane protein, is also known as folate Hydrolase 1. As such, PSMA has a role in folate metabolism and internalises ligands. It is overexpressed on most prostate cancers and exploited as a target for the treatment of patients with CRPC (133). PSMA is commonly targeted with radiolabelled urea-based ligands particularly [^177^Lu]Lu-PSMA-617 (134). Last-line salvage treatment of patients with bone involvement CRPC demonstrated that [^177^Lu]Lu-PSMA-617 improves survival and is well tolerated (134). However, more than 50% of patients with CRPC do not show biochemical response (a 50% reduction in PSA) to [^177^Lu]Lu-PSMA-617 (135). Refractory patients can receive PSMA-ligands labelled with α-emitter radiometals such as ^225^Ac (see section on α-emitters). Interestingly, a systematic review of several trials (136) identified that pretreatment with [^177^Lu]Lu-PSMA-617 in some patients may induce resistance to treatment with [^225^Ac]Ac-PSMA-617.

Several normal tissues, including the salivary gland, also express PSMA attracting binding by radiolabelled PSMA ligands, which can result in loss of salivary function resulting in xerostomia (dry mouth) (137). This side effect is particularly frequent in patients receiving PSMA ligands labelled with α-emitters (137). Mitigation can be achieved by the co-administration of mono-sodium glutamate (MSG), but xerostoma is considered a dose-limiting toxicity for patients receiving radiolabelled PSMA-targeted ligands (137). A phase I clinical trial of [^225^Ac]Ac-labelled J591 reduces xerostomia and nephrotoxicity in metastatic castration-resistant PC (mCRPC) patients associated with radiolabelled PSMA-617 (138).

## Strategies to improve the efficacy of TRT

### Use of high linear energy transfer alpha emitters

Patients with prostate cancer where the disease has spread to bone, in the absence of visceral metastasis, may receive the bone-seeking agent radium-223 dichloride, [^223^Ra]RaCl_2_, Xofigo^®^ (139). Radium is a calcium mimetic, and as such, ^223^Ra is concentrated in regions of bone turnover including adjacent to sites where metastatic cancers have established (139). Xofigo^®^, the only α-emitter radiopharmaceutical to be clinically approved, is very effective at palliation of bone pain and has been shown to improve overall survival of patients with metastatic castration resistant prostate cancer (140).

Some patients with mCRPC can become resistant to ligands radiolabelled with β-emitters such as ^177^Lu-PSMA but demonstrate response to α-emitters such as [^225^Ac]Ac-PSMA (18, 141). A phase I dose-escalation trial is currently underway to establish an optimal amount of activity for response that can be administered (141). Other [^225^Ac]-labelled therapeutics include [^225^Ac]Ac-DOTATOC and [^225^Ac]Ac-DOTA-substance-P, which have proven to be beneficial in patients with neuroendocrine tumours and gliomas, respectively (18).

Thorium-227 (^227^Th), which decays to ^223^Ra, can be chelated with octadentate 3,2-hydroxypyridinone (3,2-HOPO). Several conjugates of [^227^Th]Th-HOPO have been developed to target CD22-positive B cell cancers and CD33-positive leukaemia, and solid tumours overexpressing renal cell cancer antigen CD70 and membrane-anchored glycoprotein mesothelin in mesothelioma (142).

An α-decay event results in high-energy recoil (approximately 2% of the α-particle emission energy), which is sufficient to breach the integrity of the carrier radiopharmaceutical (143). Consequent release and free movement of the daughter species outside the tumour can result in irradiation of non-tumour tissue if the daughter undergoes further decays, sometime after the initial event, and contribute to toxicity (143).

^225^Ac (t_1/2_ = 10 d, E = 6 MeV) produces six predominant radionuclide daughters in the decay cascade to stable bismuth-209 (^209^Bi) two high-energy gamma emissions, of which ^213^Bi 440 keV is used for imaging (144). The ^225^Ac radionuclide daughters escape and circulate through the body and accumulate in different organs, and renal toxicity from released ^213^Bi is the major concern (145). Some success with nanostructures to contain radionuclide daughters after α-emission has been achieved reducing release (146). Toro-González et al. (147) encapsulated [^225^Ac]Ac^3+^ chelated by a lipophilic 2,9-bis-lactam-1,10-phenanthroline ligand in poly(lactic-co-glycolic acid) (PLGA) nanoparticles, a biocompatible delivery platform used for drug delivery. Encapsulation within 155-nm PLGA nanoparticles was found to decrease the release of daughter species [^221^Fr]Fr^+^ and [^213^Bi]Bi^3+^ but only by approximately 50%. Karpov et al. (148) modified silica nanoparticles (SiO_2_ NPs) with metallic shells composed of titanium dioxide (TiO_2_) and gold (Au) nanostructures of 110 nm in size. *In vivo* and *in vivo* studies demonstrated that the metallic surface coating of SiO_2_ NPs promotes an enhanced sequestering of radionuclides (^225^Ac and its daughter isotopes) compared with nonmodified SiO_2_. However, clearance of these large nanoparticles is hepatobiliary, which is undesirable due to the radiosensitivity of the intestinal tract.

^211^At decays with the release of a single α-emission. Feng et al. (149) have demonstrated good tolerability of [^211^At]At-labelled PSMA ligands YF2 and L3-Lu in mice bearing xenografts, derived from prostate cancer cells. Laszlo et al. (86) developed anti-CD123+ targeting ^211^At-labelled antibodies, which extended the survival of mice bearing CD123+-expressing xenografts.

^212^Pb initially undergoes β-decay to ^212^Bi, which then decays by α-emission. The initial β-decay is accompanied by a high yield of conversion electrons and a cascade of Auger electrons converting the oxidation state of Bi to between Bi^4+^ to Bi^7+^, which has been shown to result in up to 40% release of daughter [^212^Bi]Bi from chelation (150). However, preclinical work has demonstrated retention of a significant proportion of the disassociated [^212^Bi] in the tumour environment (150).

Moving forward with radiopharmaceuticals carrying α-emitters including ^225^Ac and ^212^Pb that release unchelated active daughters is likely to be challenging (151). Encapsulation in nanoparticles is an approach being explored to contain daughter species released by long-lived α-emitters, but due to the high recoil energy during α-decay, these need to be relatively large (~100 nm) (152). The large size of these particles results in hepatobiliary excretion and subsequent gut exposure (153). A further consideration with release of multiple daughter species is dosimetry. Encouragingly, a study has demonstrated that dual window planar imaging can discriminate ^223^Ra from ^227^Th in patients receiving ^223^Ra treatment (154), which enables accurate dosimetry to be determined. Clinical studies have demonstrated benefit and low toxicity of longer-lived α-emitters for example ^225^Ac to patients with advanced treatment refractory mCRPC (155). Exploration of α-emitting radionuclides towards the end of decay chains including ^212^Bi and ^213^Bi (t_1/2_s ~1 h) have demonstrated, at least preclinically good tumour control. However, the therapeutic index from [^213^Bi]Bi-PSMA-617 was reported to be lower than for [^225^Ac]Ac-PSMA-617 (156).

## Pre-targeting approaches to radionuclide delivery

**Figure 3:**
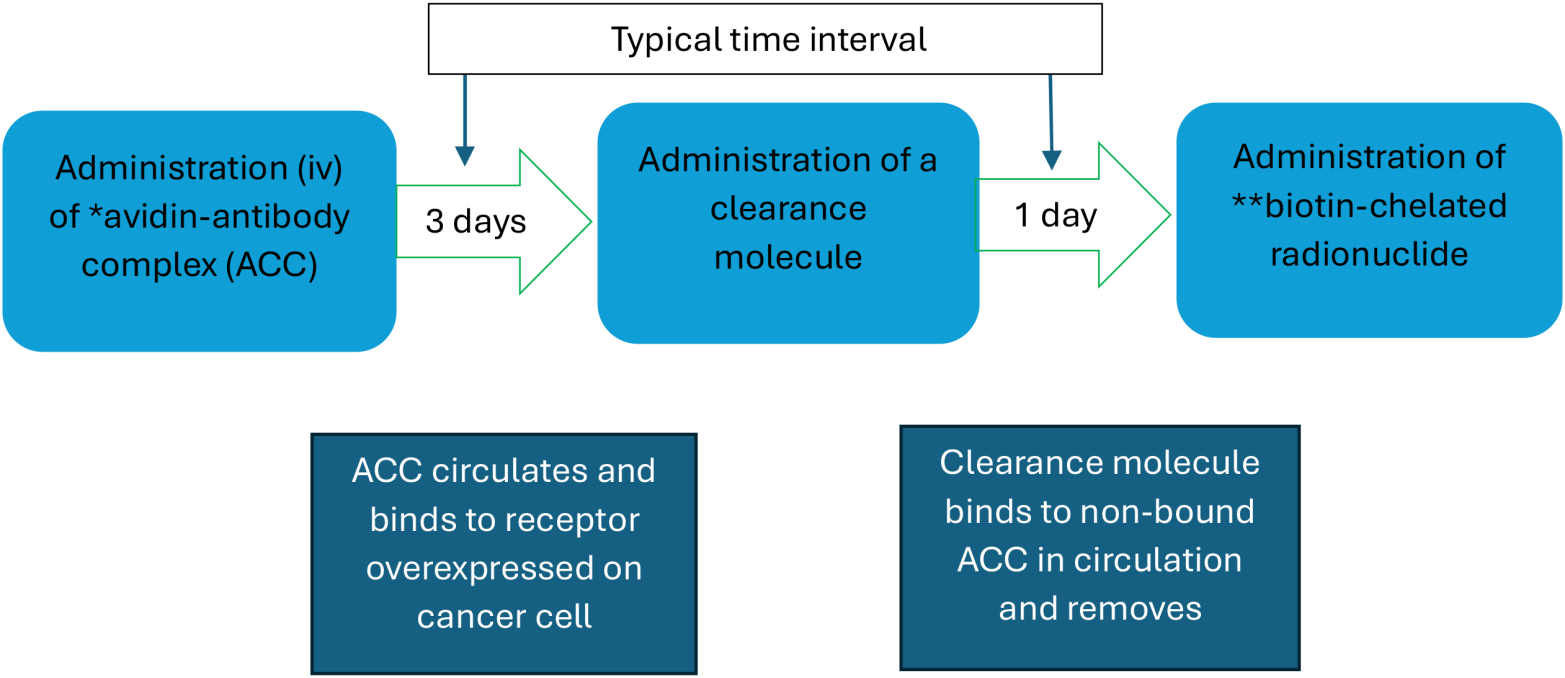
Multistep pre-targeted procedure. *Avidin and **biotin have a strong mutual affinity.

Antibody-targeted TRT radiopharmaceuticals are larger than the renal membrane and consequently have long blood clearance times, which can result in high levels of normal tissue radiation exposure (157). Pre-targeting is the initial administration of a complex consisting of an antibody conjugated, traditionally with avidin (part of an affinity pair with biotin) (see **Figure 3**). Following an interval of several days for tumour targeting and blood clearance of non-bound complex, the patient receives a radionuclide biotin conjugate, which is rapidly cleared from the circulation due to its small size (~1 kDa).

Alternative affinity pairing systems include the Diels–Alder click reaction between tetrazine (Tz) and trans-cyclooctene (TCO) (158). The TCO-modified antibody binds with the radiolabelled Tz-substituted effector poly-L-Lysine. This system has been demonstrated to carry ^211^At for targeted alpha therapy and radioiodine for imaging. Poly-L-lysine was functionalised with a prosthetic group, for the attachment of both radiohalogens and tetrazine (159). As for direct targeting, pre-targeted approaches to TRT delivery require imaging using the same format of delivery system so that the imaging biomarkers accurately demonstrate tumour uptake of the therapeutic.

The theragnostic capability of the Tz/TCO pre-targeting system with the copper isotopes [^64^Cu]- and [^67^Cu]-radiolabelled Tz ([^64/67^Cu]Cu-MeCOSar-Tz) was evaluated in a murine model of colorectal cancer (160). Mice pre-administered with the huA33 antibody modified with TCO (huA33-TCO) were treated 72 h later with [^64/67^Cu]Cu-MeCOSar-Tz. Tumour uptake of [^64^Cu] predicted response to treatment with [^67^Cu]Cu-MeCOSar-Tz given either in a single dose or fractionated. Theragnostic approaches using the Tz/TCO system has also been examined using a self-assembling and disassembling molecular system for treating glioblastoma using murine models (161).

## Combination of TRT with immune activation and radiosensitisation

The primary mechanism of cell death induced by ^223^Ra is by induction of DNA damage particularly double-strand breaks (DSBs) (162). Other mechanisms that may contribute to the therapeutic efficacy of Xofigo^®^ include immunogenic mechanisms (163). DNA damage can activate the stimulator of interferon gene (STING) signalling pathway activating NLRP3-dependent pyroptosis, a typical form of immunogenic cell death, to enhance antitumor immune response. This may be an important mechanism of tumour control induced by ^223^Ra-irradiation (163). Biomarkers that can guide selection of patients for treatment with PD-1/PD-L1 inhibitors include PD-1/PD-L1 expression and mismatch repair deficiency (164).

TRT treatments may enhance immune response through enhancement of infiltration of CD4^+^ and CD8^+^ T cells (165). Zboralski et al. (166) have shown that combined treatment of mice bearing FAP-expressing xenografts with a [^177^lu]Lu-labelled FAP inhibitor ([^177^Lu]Lu-FAP-2287) and a PD-1 inhibitor increased recruitment of tumour-infiltrating CD8(+) T cells.

Clinical trials combining immune checkpoint inhibitors (ICIs) with TRT are underway including the STARLITE 2 Phase 2 trial of patients with clear cell renal carcinoma treated with the ICI, nivolumab, and [^177^Lu]Lu-girentuximab, which is an anti-carbonic anhydrase IX antibody (167).

Interestingly, the type of radionuclide influences the synergistic therapeutic effect of TRT with PD-1 inhibitors. Compared with monotherapy, combination treatments of PD-1 inhibitors with [^213^Bi]-anti-melanin and to a lesser extent [^177^lu]Lu-anti-melanin, but not [^225^Ac]-anti-melanin, increased the treatment response of melanomas (168).

Chimeric antigen receptor T cells (CART) are T lymphocytes that have been taken from a patient and reprogrammed to identify and attack their cancers (169). CARTs are relatively ineffective against solid tumours, but this can be increased by exposure of the cancers to radiation (170). Sodji et al. (170) compared the effector (cytotoxic activity against GD2 expressing human neuroblastoma (CHLA-20) and M21 melanoma cells) and viability of anti-GD2 CART cells after exposure to different doses from ^225^Ac or ^177^Lu. Radiation enhanced the cytotoxic activity of these CAR T cells against CHLA-20 and M21 independent of dose tested and type of radionuclide. ^225^Ac was more toxic than ^177^Lu to anti-GD2 CAR T cells, suggesting that ^177^Lu-based TRT may be preferred over ^225^Ac-based TRT to enhance CAR T activity.

Radiosensitisers are commonly used to improve the therapeutic efficacy of external beam radiotherapy (171). Radio-sensitisation can be mediated through many mechanisms including chemotherapy and inhibition of DNA damage response proteins such as ATM and DNA-PKcs and DNA repair enzymes, e.g., topoisomerase I and PARP inhibitors. Phase 1 trials of radiotherapy with the potent ATM/DNA-PKcs inhibitor XRD-0394 has recently reported favourable results (172). Beneficial patient outcomes of radiosensitisers with radiation therapy are a strong rationale for combination treatments with molecular radiotherapy.

Antimetabolite chemotherapy drugs including 5-fluorouracil (5FU) and capecitabine are commonly used in combination with radiotherapy (171). Combination treatments of patients with neuroendocrine tumours with [^177^Lu]Lu-DOTATATE and 5FU or capecitabine are well tolerated (173). Some studies have demonstrated that combining TRT with capecitabine improves patient outcome (174).

**Figure 4:**
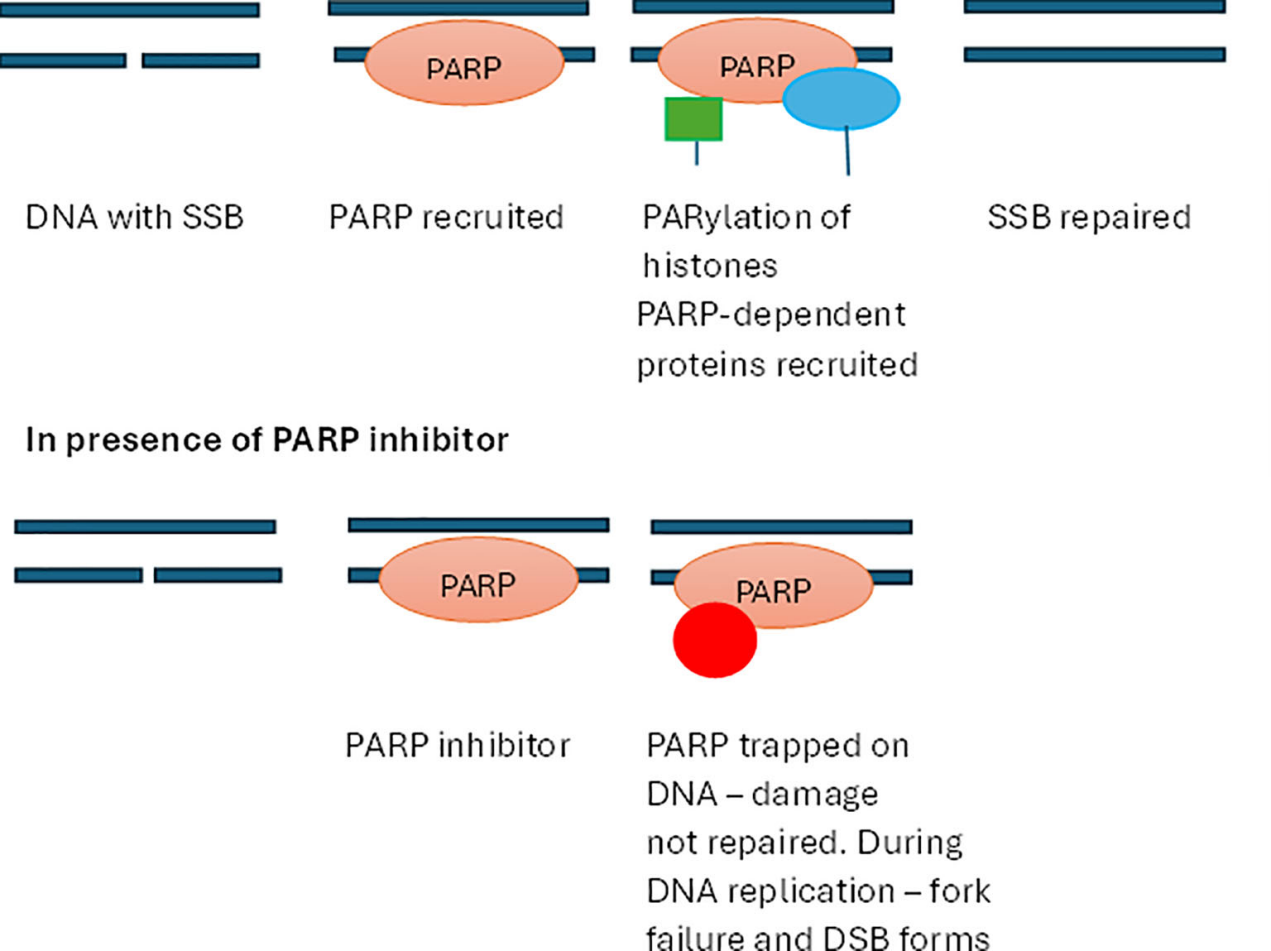
Mode of action of poly ADP-ribose and its inhibition during repair of single-strand DNA breaks.

Response to radiation-based treatments is countered by DNA repair mechanisms (175). Targeting DNA repair enzymes is an established form of radiosensitisation (176). Poly(ADP-ribose) polymerase 1 (PARP-1) is essential in DNA single-strand break (SSB) repair (see **Figure 4**). Several PARP inhibitors (PARPi) are now clinically approved (176). These bind to PARP/SSB complexes formed on damaged DNA preventing repair and disengagement of the repair complex, which in turn results in double-strand break (DSB) formation. BRCA1/2 mutant cancers have a limited capacity to repair DSBs. PARP inhibitors are particularly effective for patients with mutant BRCA1/2 cancers (177). Combination treatment of a murine model of a triple-negative breast cancer with the radiolabelled FAP inhibitor [^177^Lu]Lu-DOTAGA.(SA.FAPi)(2) and PARP inhibitor olaparib increased therapeutic efficacy over [^177^Lu]Lu-DOTAGA.(SA.FAPi)(2) alone (178). Clinical trials (early phase) assessing combination PARPi + [177Lu]Lu-DOTATATE for pancreatic and metastatic NENs are ongoing (173).

Tyrosine kinases (TK) are components of receptors and cell signalling pathways, many of which induce cell growth and proliferation. Anticancer treatments include TK inhibitors (TKI) (173). Some TKIs are multi-TK inhibitor, such as sunitinib, and can inhibit angiogenesis as well as cancer cell growth. Early phase trials of combination treatments of sunitinib and other TK inhibitors with NEN-targeted TRT are ongoing (173).

To discover potential strategies for sensitisation to TRT, Qin et al. (179) examined protein activation in cholecystokinin B receptor expressing A431 cancer cells treated with a ^225^Ac-labeled minigastrin analogue ([^225^Ac]Ac-PP-F11N) using proteomics and phospho-proteomics. They identified several upregulated proteins associated with carcinogenesis and DNA repair pathways including histone deacetylases (HDAC) for which there is an FDA-approved inhibitor, SAHA. Treatment of A431/CCKB cells with SAHA and [^225^Ac]Ac-PP-F11N was synergistic over treatment with each alone delivering increased DSB formation and tumour cell kill.

Many studies have demonstrated that hyperthermia can improve response to radiotherapy (180). Heating is delivered through several techniques including ultrasound to raise local temperature to 40 °C-43°C. Mechanisms responsible for the beneficial effect of hyperthermia include improved blood flow, inhibition of DNA repair, and immune activation (176). Gold nanoparticles have the property of near IF light (NIR) to heat conversion, known as the photothermal effect (PT). When incorporated into tumour tissue and activated by NIR, AuNPs can achieve localised and targeted hyperthermia (181). Simón et al. (181) demonstrated that PT, delivered with NIR irradiation of xenografts in mice administered with AuNPs, improved the therapeutic efficacy of [^177^Lu]Lu-DOTA-TATE PRRT against SSTR-expressing cancers in mice.

DNA irradiated with ionising radiation is damaged either by direct interaction with radiation or via the formation of free radicals particularly reactive oxygen species (ROS) (182). Photodynamic therapy (PDT) is mediated by the formation of ROS from molecular oxygen by the transfer of energy from photosensitisers excited by light at specific wavelengths (183). Generated ROS will then contribute to DNA damage. A precursor of the photosensitiser, protoporphyrin IX (PpIX), 5-aminolevulinic acid (5-ALA), accumulates in cancer tissue (184). Jo et al. (184) demonstrated that photodynamic therapy can be elicited using Cerenkov luminescence energy transfer (CLET) from the decay of ^64^Cu to PpIX in mice administered with [^64^Cu]Cu-DOTA-trastuzumab and 5-ALA for high-precision PDT of HER-2-overexpressing cancer.

Gold nanoparticles can enhance radiosensitivity (185). Cysteine functionalised glutathione-coated gold nanoparticles, l-Cys-GSH-AuNP, aggregate in the acidic conditions in lysosomes facilitating lysosomal accumulation on internalisation by cancer cells. PET imaging of xenograft-bearing mice injected with [^68^Ga]Ga-l-Cys-GSH-AuNP demonstrated long-term tumour accumulation and increased response to external beam radiotherapy (185).

## Discussion

TRT is a relatively recent addition to the anticancer treatment repertoire. In the last few years, the phase 3 NETTER and VISION trials of [^177^Lu]-radiolabelled TRT agents for neuroendocrine cancer and prostate cancer respectively have reported beneficial outcomes in terms of improved progression free survival (PFS) (NETTER) (124) and overall survival and PFS (VISION) (162). These findings have greatly increased interest in TRT especially for prostate cancer treatment and expansion to other cancer types. This has brought into focus the requirement for reliable supplies of medical radionuclides and the need for an expansion in production facilities.

Pretreatment imaging of candidate patients is essential to demonstrate suitability for TRT and inform on dosimetry (48). Ideally, imaging should be carried out with an imaging component of a theragnostic pair to provide biodistribution and dosimetry information that will most accurately predict the pharmacokinetics of the therapeutic (49).

Combining TRT with other treatments can improve efficacy increasing long-term benefit or allow for lower radioactive doses reducing toxicity and helping to minimise the number of patients who withdraw from treatment. Radiosensitisers are commonly used with external beam radiotherapy (EBRT) to improve response but could be applied alongside TRT (186). Predictive biomarkers can be used clinically to inform on radiosensitiser use for patients with hypoxic, and therefore radioresistant, cancers (187).

Approximately 25% of patients with lymphoma treated with [^90^Y]Y- 90 Y-ibritumomab tiuxetan (^90^Y-IT, Zevalin, Acrotech Biopharma) demonstrate complete long-term remission (119). Many patients with mCRPC patients benefit from [^177^Lu]PSMA-617, but approximately 30% do not respond. These findings illustrate the need for predictive biomarkers based on intrinsic tumour biology to stratify patients for treatment modalities. Several cancer types have been shown to be differentiable into molecular subtypes (188). The molecular subtype is associated with response or resistance to different treatment types; for example, some subtypes of bladder cancer are resistant to chemotherapy but sensitive to other treatments (188). Studies that stratify TRT response by molecular subtype may help to define which patients may benefit from TRT or require sensitisation. Other approaches include the use of intrinsic radiosensitivity gene expression signatures as developed for external beam radiotherapy (189). Studies have examined TRT-upregulated genes which may help to identify candidate signature genes (190). These approaches will require multiple large patient cohorts for validation. Applying biomarkers to combination treatments will require an assessment of suitability of the patient for each treatment type using multiple biomarkers.

Some patients, refractory to β-emitter-radiolabelled PSMA and somatostatin ligands, have demonstrated beneficial response to ligands radiolabelled with α-emitters such as ^225^Ac (125). Daughter decay products from the longer-lived α-emitters like ^225^Ac tend to be released from their chelate and associated with normal tissue toxicity. Fortunately, toxicity associated with [^225^Ac] Ac-PSMA-617 treatment of patients with mCRPC is considered low (155). However, supply of ^225^Ac is a serious issue. A further limitation in some European countries is the requirement for hospital confinement for several days post-treatment reducing the number of patients who can be treated at any one time.

Inhomogeneous dose distribution is a common problem with TRT due to the heterogeneity in target expression between cancer cells, even within the same tumour, and regions of poor blood perfusion due to the rudimentary nature of the tumour vasculature (191). Dose distribution inhomogeneity may result in undertreatment of some cancer cells and inevitable tumour recurrence. The crossfire effect, which is the deposition of energy emitted from a radionuclide into distant cells, particularly from β-emitters, helps to even out dose across tumours. Combinations of β-emitters with different energies will result in a greater spread of dose and may further help in overcoming dose inhomogeneities (127).

Hypoxic cancer cells which are resistant to low LET radiation but sensitive to high LET radiation tend to be distributed as single cells or small groups of cells (192). Alpha-particles with their short range and highly damaging characteristics within a few cell diameters are ideal for targeting hypoxic cells in tumours.

TRT is an exciting approach to cancer treatment with many variation possibilities including use of multiple radionuclides to improve dose distribution, combined with other treatments to synergise treatment efficacy to bring about its optimisation. Validated biomarkers to identify patients most likely to benefit or conversely who may not benefit from TRT are crucial. The capacity to produce and deliver medically radionuclides particularly, ^177^Lu, for treatment is essential to ensure that these effective treatments can be expanded to fulfil the predicted demand.

## References

[1] Sawin CT Becker DV. Radioiodine and the treatment of hyperthyroidism: the early history. Thyroid. (1997) 7:163–76. doi: 10.1089/thy.1997.7.163, PMID: 9133679

[2] Parmentier C. Use and risks of phosphorus-32 in the treatment of polycythaemia vera. Eur J Nucl Med Mol Imaging. (2003) 30:1413–7. doi: 10.1007/s00259-003-1270-6, PMID: 12955483

[3] Sindhu KK Nehlsen AD Stock RG. Radium-223 for metastatic castrate-resistant prostate cancer. Pract Radiat Oncol. (2022) 12:312–6. doi: 10.1016/j.prro.2022.03.004, PMID: 35717046

[4] Dekempeneer Y Keyaerts M Krasniqi A Puttemans J Muyldermans S Lahoutte T. Targeted alpha therapy using short-lived alpha-particles and the promise of nanobodies as targeting vehicle. Expert Opin Biol Ther. (2016) 16:1035–47. doi: 10.1080/14712598.2016.1185412, PMID: 27145158 PMC4940885

[5] Sneddon D Cornelissen B. Emerging chelators for nuclear imaging. Curr Opin Chem Biol. (2021) 63:152–62. doi: 10.1016/j.cbpa.2021.03.001, PMID: 34051509

[6] Filippi L Bagni O Nervi C. Aptamer-based technology for radionuclide targeted imaging and therapy: a promising weapon against cancer. Expert Rev Med Devices. (2020) 17:751–8. doi: 10.1080/17434440.2020.1796633, PMID: 32669004

[7] Tsai WK Zettlitz KA Dahlbom M Reiter RE Wu AM. Evaluation of [131I]I- and [177Lu]Lu-DTPA-A11 minibody for radioimmunotherapy in a preclinical model of PSCA-expressing prostate cancer. Mol Imaging Biol. (2020) 22:1380–91. doi: 10.1007/s11307-020-01518-4, PMID: 32661830 PMC7688013

[8] Aerts A Impens NR Gijs M D’Huyvetter M Vanmarcke H Ponsard B. Biological carrier molecules of radiopharmaceuticals for molecular cancer imaging and targeted cancer therapy. Curr Pharm Des. (2014) 20:5218–44. doi: 10.2174/1381612819666140110114902, PMID: 24606796

[9] Hu XW Li DD Fu YJ Zheng JS Feng ZL Cai J. Advances in the application of radionuclide-labeled HER2 affibody for the diagnosis and treatment of ovarian cancer. Front Oncol. (2022) 12:917439. doi: 10.3389/fonc.2022.917439, PMID: 35785201 PMC9240272

[10] Boschi A Uccelli L Martini P. A picture of modern Tc-99m radiopharmaceuticals: production, chemistry, and applications in molecular imaging. Appl Sci. (2019) 9:2526. doi: 10.3390/app9122526

[11] Braune A Oehme L Freudenberg R Hofheinz F van den Hoff J Kotzerke J. Comparison of image quality and spatial resolution between 18F, 68Ga, and 64Cu phantom measurements using a digital Biograph Vision PET/CT. EJNMMI Phys. (2022) 9:58. doi: 10.1186/s40658-022-00487-7, PMID: 36064989 PMC9445107

[12] Rudd SE Noor A Morgan KA Donnelly PS. Diagnostic positron emission tomography imaging with zirconium-89 desferrioxamine B squaramide: from bench to bedside. Acc Chem Res. (2024) 57:1421–33. doi: 10.1021/acs.accounts.4c00092, PMID: 38666539

[13] Sgouros G Bodei L McDevitt MR Nedrow JR. Radiopharmaceutical therapy in cancer: clinical advances and challenges. Nat Rev Drug Discov. (2020) 19:589–608. doi: 10.1038/s41573-020-0073-9, PMID: 32728208 PMC7390460

[14] Miller C Klyuzhin I Chaussé G Brosch-Lenz J Koniar H Shi K. Impact of cell geometry, cellular uptake region, and tumour morphology on 225Ac and 177Lu dose distributions in prostate cancer. EJNMMI Phys. (2024) 11:97. doi: 10.1186/s40658-024-00700-9, PMID: 39570450 PMC11582247

[15] Nunes BS Rodrigues ERF Fruscalso JAP Nunes RP Bonatto A Alva-Sanchez MS. Highly enriched uranium-free medical radioisotope production methods: an integrative review. App Sci Basel. (2022) 12:12569. doi: 10.3390/app122412569

[16] Vogel WV van der Marck SC Versleijen MWJ. Challenges and future options for the production of lutetium-177. Eur J Nucl Med Mol Imag. (2021) 48:2329–35. doi: 10.1007/s00259-021-05392-2, PMID: 33974091 PMC8241800

[17] Nawar MF Türler A. New strategies for a sustainable 99mTc supply to meet increasing medical demands: Promising solutions for current problems. Front Chem. (2022) 10:926258. doi: 10.3389/fchem.2022, PMID: 35936080 PMC9355089

[18] Hassan M Bokhari TH Lodhi NA Khosa MK Usman M. A review of recent advancements in Actinium-225 labeled compounds and biomolecules for therapeutic purposes. Chem Biol Drug Design. (2023) 102:1276–92. doi: 10.1111/cbdd.14311, PMID: 37715360

[19] Jalloul W Ghizdovat V Stolniceanu CR Ionescu T Grierosu IC Pavaleanu I. Targeted alpha therapy: all we need to know about 225Ac’s physical characteristics and production as a potential theragnostic radionuclide. Pharm (Basel). (2023) 16:1679. doi: 10.3390/ph16121679, PMID: 38139806 PMC10747780

[20] Chakravarty R Shetty P Nair KV Rajeswari A Jagadeesan KC Sarma HD. Reactor produced [64Cu] CuCl2 as a PET radiopharmaceutical for cancer imaging: From radiochemistry laboratory to nuclear medicine clinic. Ann Nucl Med. (2020) 34:899–910. doi: 10.1007/s12149-020-01522-2, PMID: 33048309

[21] Dellepiane G Casolaro P Mateu I Scampoli P Braccini S. Alternative routes for 64Cu production using an 18 MeV medical cyclotron in view of theragnostic applications. Appl Radiat Isot. (2023) 191:110518. doi: 10.1016/j.apradiso.2022.110518, PMID: 36327610

[22] Broder BA Bhuiyan MP Freifelder R Zhang HJ Kucharski A Makinen MW. Preliminary investigation of 48V-labeled VO(acac) 2 for cancer imaging: An initial proof-of-concept study. Appl Radiat Isotopes. (2022) 186:110270. doi: 10.1016/j.apradiso.2022.110270, PMID: 35569262

[23] Meier JP Zhang HJ Freifelder R Bhuiyan M Selman P Mendez M. Accelerator-based production of scandium radioisotopes for applications in prostate cancer: toward building a pipeline for rapid development of novel theragnostics. Molecules. (2023) 28:6041. doi: 10.3390/molecules28166041, PMID: 37630292 PMC10458970

[24] Chakravarty R Banerjee D Chakraborty S. Alpha-induced production and robust radiochemical separation of 43Sc as an emerging radiometal for formulation of PET radiopharmaceuticals. App Radiat Isotopes. (2023) 199:110921. doi: 10.1016/j.apradiso.2023.110921, PMID: 37413711

[25] van der Meulen NP Hasler R Talip Z Grundler PV Favaretto C Umbricht CA. Developments toward the Implementation of 44Sc Production at a Medical Cyclotron. Molecules. (2020) 25:4706. doi: 10.3390/molecules25204706, PMID: 33066650 PMC7587374

[26] Ali W Hussain M Amjad N. Evaluation of the nuclear reaction cross sections via proton induced reactions on 72Ge and 76Se to produce 72As: A potential entrant for the theragnostic pairs. App Radiat Isotopes. (2021) 168:109507. doi: 10.1016/j.apradiso.2020.109507, PMID: 33317890

[27] Oroujeni M Xu TQ Gagnon K Rinne SS Weis J Garousi J. The use of a non-conventional long-lived gallium radioisotope 66Ga improves imaging contrast of EGFR expression in Malignant tumours using DFO-ZEGFR:2377 affibody molecule. Pharmaceutical. (2024) 13:292. doi: 10.3390/pharmaceutics13020292, PMID: 33672373 PMC7926986

[28] Patra S Ghosh S Singh K Dutta B Chakraborty A Gamre N. Accelerator production, radiochemical separation and nanoradiopharmaceutical formulation using 69Ge: A next generation PET probe. J Drug Deliv Sci Technol. (2024) 91:105204. doi: 10.1016/j.jddst.2023.105204

[29] Lamparter D Hallmann B Hänscheid H Boschi F Malinconico M Samnick S. Improved small scale production of iodine-124 for radiolabeling and clinical applications. App Radiat Isotopes. (2018) 140:24–8. doi: 10.1016/j.apradiso.2018.06.014, PMID: 29936272

[30] Zhang Y Hong H Cai W. PET tracers based on Zirconium-89. Curr Radiopharm. (2011) 4:131–9. doi: 10.2174/1874471011104020131, PMID: 22191652 PMC3246366

[31] Costa OD Barcellos H Matsuda H Sumiya LD Junqueira FC Matsuda MMN. A new 124Xe irradiation system for 123I production. Appl Radiat Isotopes. (2023) 200:110926. doi: 10.1016/j.apradiso.2023.110926, PMID: 37459684

[32] Gao J Liao Z Liu W Hu Y Ma H Xia L. Simple and efficient method for producing high radionuclidic purity 111In using enriched 112Cd target. Appl Radiat Isot. (2021) 176:109828. doi: 10.1016/j.apradiso.2021.109828, PMID: 34166947

[33] Poorbaygi H Roozbahani A Moradi K. Preparation of 90Y by a 90Sr-90Y chromatographic generator using combined columns containing Sr resin and DGA resin for radionuclide therapy. J Radioanal Nucl Chem. (2021) 327:985–90. doi: 10.1007/s10967-020-07579-7

[34] Dash A Pillai MR Knapp FF Jr. Production of (177)Lu for targeted radionuclide therapy: available options. Nucl Med Mol Imaging. (2015) 49:85–107. doi: 10.1007/s13139-014-0315-z, PMID: 26085854 PMC4463871

[35] Abou DS Picketta J Mattson JE Thorek DLJ. A Radium-223 microgenerator from cyclotron-produced trace Actinium-227. Appl Radiation Isotopes. (2017) 119:36–42. doi: 10.1016/j.apradiso.2016.10.015, PMID: 27835737 PMC5136344

[36] Ondrák L Fialová KO Sakmár M Vlk M Bruchertseifer F Morgenstern A. Development of 225Ac 213Bi generator based on α-ZrP-PAN composite for targeted alpha therapy. Nucl Med Biol. (2024) 132:108909. doi: 10.1016/j.nucmedbio.2024.108909, PMID: 38599144

[37] Mcintosh LA Burns JD Tereshatov EE Muzzioli R Hagel K Jinadu NA. Production, isolation, and shipment of clinically relevant quantities of astatine-211: A simple and efficient approach to increasing supply. Nucl Med Biol. (2023) 126:108387. doi: 10.1016/j.nucmedbio.2023.108387, PMID: 37837782

[38] Das T Pillai MR. Options to meet the future global demand of radionuclides for radionuclide therapy. Nucl Med Biol. (2013) 40:23–32. doi: 10.1016/j.nucmedbio.2012.09.007, PMID: 23116551

[39] Van de Voorde M Duchemin C Heinke R Lambert L Chevallay E Schneider T. Production of Sm-153 with very high specific activity for targeted radionuclide therapy. Front Med. (2021) 8:675221. doi: 10.3389/fmed.2021.675221, PMID: 34350194 PMC8326506

[40] Lee JY Chae JH Hur MG Yang SD Kong YB Lee J. Theragnostic 64Cu/67Cu radioisotopes production with RFT-30 cyclotron. Front Med. (2022) 9:889640. doi: 10.3389/fmed.2022.889640, PMID: 35665337 PMC9158440

[41] Domnanich KA Müller C Benešová M Dressler R Haller S Köster U. 47Sc as useful β–emitter for the radiotheragnostic paradigm: a comparative study of feasible production routes. EJNMMI Radiopharm Chem. (2017) 2:5. doi: 10.1186/s41181-017-0024-x, PMID: 29503846 PMC5824697

[42] Snow MS Foley A Ward JL Kinlaw MT Stoner J Carney KP. High purity 47Sc production using high-energy photons and natural vanadium targets. App Rad Isotopes. (2021) 178:109934. doi: 10.1016/j.apradiso.2021.109934, PMID: 34598038

[43] Vats K Das T Sarma HD Banerjee S Pillai MRA. Radiolabeling, stability studies, and pharmacokinetic evaluation of thulium-170-labeled acyclic and cyclic polyaminopolyphosphonic acids. Cancer Biother Radiopharmaceut. (2013) 28:737–45. doi: 10.1089/cbr.2013.1475, PMID: 23931111

[44] Saghez BS Yang H Radchenko V. High separation factor, high molar activity, and inexpensive purification method for the production of pure 165Er. Inorg Chem. (2024) 63:5330–40. doi: 10.1021/acs.inorgchem.3c03166, PMID: 38324916

[45] Renaldin E Dellepiane G Braccini S Sommerhalder A Zhang H van der Meulen NP. Study of thulium-167 cyclotron production: a potential medically-relevant radionuclide. Front Chem. (2023) 11:1288588. doi: 10.3389/fchem.2023.1288588, PMID: 37927558 PMC10620610

[46] Vosoughi S Rovias MRA Rahiminezhad A Novin FB Yousefi K Sardjono Y. Production assessment of 195mPt in Tehran research reactor. J Radioanalytic Nucl Chem. (2023) 332:2989–94. doi: 10.1007/s10967-023-09008-x

[47] Naskar N Lahiri S. Theragnostic terbium radioisotopes: challenges in production for clinical application. Front Med (Lausanne). (2021) 8:675014. doi: 10.3389/fmed.2021.675014, PMID: 34136508 PMC8200528

[48] O’Neill E Cornelissen B. Know thy tumour: Biomarkers to improve treatment of molecular radionuclide therapy. Nucl Med Biol. (2022) 108-109:44–53. doi: 10.1016/j.nucmedbio.2022.02.004, PMID: 35276447

[49] Barca C Griessinger CM Faust A Depke D Essler M Windhorst AD. Expanding theranostic radiopharmaceuticals for tumor diagnosis and therapy. Pharm (Basel). (2021) 15:13. doi: 10.3390/ph15010013, PMID: 35056071 PMC8780589

[50] Artigas C Mileva M Flamen P Karfis I. Targeted radionuclide therapy: an emerging field in solid tumours. Curr Opin Oncol. (2021) 33:493–9. doi: 10.1097/CCO.0000000000000762, PMID: 34183491

[51] Ferris T Carroll L Jenner S Aboagye EO. Use of radioiodine in nuclear medicine-A brief overview. J Labelled Comp Radiopharm. (2021) 64:92–108. doi: 10.1002/jlcr.3891, PMID: 33091159

[52] Ioannidis I Lefkaritis G Georgiades SN Pashalidis I Kontoghiorghes GJ. Towards clinical development of scandium radioisotope complexes for use in nuclear medicine: encouraging prospects with the chelator 1,4,7,10-tetraazacyclododecane-1,4,7,10-tetraacetic acid (DOTA) and its analogues. Intl J Mol Sci. (2024) 25:5954. doi: 10.3390/ijms25115954, PMID: 38892142 PMC11173192

[53] Simms ME Li ZY Sibley MM Ivanov AS Lara CM Johnstone TC. PYTA: a universal chelator for advancing the theragnostic palette of nuclear medicine. Chem Sci. (2024) 15:11279–82. doi: 10.1039/d3sc06854d, PMID: 39055008 PMC11268510

[54] Franchi S Madabeni A Tosato M Gentile S Asti M Orian L. Navigating through the coordination preferences of heavy alkaline earth metals: Laying the foundations for 223 Ra- and 131/135m Ba-based targeted alpha therapy and theragnostics of cancer. J Inorg Biochem. (2024) 256:112569. doi: 10.1016/j.jinorgbio.2024.112569, PMID: 38701687

[55] Gemini-Piperni S Ricci E Ilem-Özdemir D Batista BD Alencar LMR Rossi AM. Nano-hydroxyapatite radiolabeled with radium dichloride [223Ra] RaCl2 for bone cancer targeted alpha therapy: *In vitro* assay and radiation effect on the nanostructure. Colloids Surf B Biointerf. (2023) 223:113174. doi: 10.1016/j.colsurfb.2023.113174, PMID: 36746067

[56] Trencsényi G Csikos C Képes Z. Targeted radium alpha therapy in the era of nanomedicine: *in vivo* results. Int J Mol Sci. (2024) 25:664. doi: 10.3390/ijms25010664, PMID: 38203834 PMC10779852

[57] Bailey TA Wacker JN An DD Carter KP Davis RC Mocko V. Evaluation of 134Ce as a PET imaging surrogate for antibody drug conjugates incorporating 225Ac Nucl. Med Biol. (2022) 110:28–36. doi: 10.1016/j.nucmedbio.2022.04.007, PMID: 35512517

[58] Garnuszek P Karczmarczyk U Maurin M Sikora A Zaborniak J Pijarowska-Kruszyna J. PSMA-D4 radioligand for targeted therapy of prostate cancer: synthesis, characteristics and preliminary assessment of biological properties. Inter J Mol Sci. (2021) 22:2731. doi: 10.3390/ijms22052731, PMID: 33800517 PMC7962978

[59] Alati S Singh R Pomper MG Rowe SP Banerjee SR. Preclinical development in radiopharmaceutical therapy for prostate cancer. Semin Nucl Med. (2023) 53:663–86. doi: 10.1053/j.semnuclmed.2023.06.007, PMID: 37468417

[60] Böhnke N Indrevoll B Hammer S Papple A Kristian A Briem H. Mono- and multimeric PSMA-targeting small molecule-thorium-227 conjugates for optimized efficacy and biodistribution in preclinical models Eur. J Nucl Med Mol Imag. (2024) 51:669–80. doi: 10.1007/s00259-023-06474-z, PMID: 37882848 PMC10796422

[61] Saeki N Gu J Yoshida T Wu X. Prostate stem cell antigen: a Jekyll and Hyde molecule? Clin Cancer Res. (2010) 16:3533–8. doi: 10.1158/1078-0432.CCR-09-3169, PMID: 20501618 PMC2905486

[62] Huynh TT van Dam EM Sreekumar S Mpoy C Blyth BJ Muntz F. Copper-67-labeled bombesin peptide for targeted radionuclide therapy of prostate cancer. Pharmaceuticals. (2022) 15:728. doi: 10.3390/ph15060728, PMID: 35745647 PMC9229378

[63] Kanellopoulos P Lymperis E Kaloudi A de Jong M Krenning EP Nock BA. 99mTc]Tc-DB1 mimics with different-length PEG spacers: preclinical comparison in GRPR-positive models. Molecules. (2020) 25:3418. doi: 10.3390/molecules25153418, PMID: 32731473 PMC7435657

[64] Busslinger SD Tschan VJ Richard OK Talip Z Schibli R Müller C. 225Ac]Ac-SibuDAB for targeted alpha therapy of prostate cancer: preclinical evaluation and comparison with [225Ac]Ac-PSMA-617. Cancers. (2022) 14:5651. doi: 10.3390/cancers14225651, PMID: 36428743 PMC9688344

[65] Ren YN Liu TL Liu C Guo XY Wang F Zhu H. An albumin-binding PSMA ligand with higher tumor accumulation for PET imaging of prostate cancer. Pharmaceuticals. (2022) 15:513. doi: 10.3390/ph15050513, PMID: 35631340 PMC9143078

[66] El Bakkali J Doudouh A El Bardouni T Ghalbzouri TEL Yerrou R. Intercomparison of S-Factor values calculated in Zubal voxelized phantom for eleven radionuclides commonly used in targeted prostate cancer therapy. Phys Eng Sci Med. (2022) 45:1251–6. doi: 10.1007/s13246-022-01191-7, PMID: 36315382

[67] Choi YJ Park JY Cho YL Chae JR Cho H Kang WJ. *In vivo* positron emission tomography imaging for PD-L1 expression in cancer using aptamer. Biochem Biophys Res Communic. (2022) 620:105–12. doi: 10.1016/j.bbrc.2022.06.059, PMID: 35780578

[68] Leonte RA Chilug LE Serban R Mustaciosu C Raicu A Manda G. Preparation and preliminary evaluation of neurotensin radiolabelled with 68Ga and 177Lu as potential theragnostic agent for colon cancer. Pharmaceuticals. (2021) 13:506. doi: 10.3390/pharmaceutics13040506, PMID: 33917046 PMC8067721

[69] Okarvi SM Al-Jammaz I. Synthesis, radiolabeling, and preclinical evaluation of 68Ga/177Lu-labeled leuprolide peptide analog for the detection of breast cancer. Cancer Biother Radiopharm. (2022) 37:372–83. doi: 10.1089/cbr.2021.0370, PMID: 35325547

[70] Liu YS Yu SZ Xu TQ Bodenko V Orlova A Oroujeni M. Preclinical evaluation of a new format of 68Ga- and 111In-labeled affibody molecule ZIGF-1R:4551 for the visualization of IGF-1R expression in Malignant tumors using PET and SPECT. Pharmaceutics. (2022) 14:1475. doi: 10.3390/pharmaceutics14071475, PMID: 35890370 PMC9320461

[71] Broqueza J Prabaharan CB Andrahennadi S Allen KJH Dickinson R MacDonald-Dickinson V. Novel human antibodies to insulin growth factor 2 receptor (IGF2R) for radioimmunoimaging and therapy of canine and human osteosarcoma. Cancers. (2021) 13:2208. doi: 10.3390/cancers13092208, PMID: 34064450 PMC8124616

[72] Lin FC Clift R Ehara T Yanagida H Horton S Noncovich A. Peptide binder to glypican-3 as a theranostic agent for hepatocellular carcinoma. J Nucl Med. (2024) 65:586–92. doi: 10.2967/jnumed.123.266766, PMID: 38423788

[73] Labadie KP Hamlin DK Kenoyer A Daniel SK Utria AF Ludwig AD. Glypican-3-targeted 227Th α-therapy reduces tumor burden in an orthotopic xenograft murine model of hepatocellular carcinoma. J Nucl Med. (2022) 63:1033–8. doi: 10.2967/jnumed.121.262562, PMID: 34772791 PMC9258570

[74] Kadassery KJ King AP Fayn S Baidoo KE MacMillan SN Escorcia FE. H2BZmacropa-NCS: A bifunctional chelator for actinium-225 targeted alpha therapy. Bioconj Chem. (2022) 33:1222–31. doi: 10.1021/acs.bioconjchem.2c00190, PMID: 35670495 PMC9362842

[75] Kryza D Wischhusen J Richaud M Hervieu M Boumedine JS Delcros JG. From netrin-1-targeted SPECT/CT to internal radiotherapy for management of advanced solid tumors. EMBO Mol Med. (2023) 15:e16732. doi: 10.15252/emmm.202216732, PMID: 36876343 PMC10086585

[76] Wu YT Zhu H Zhang XJ Yu P Gui Y Xu ZH. Synthesis and evaluation of [99mTc]TcAMD3465 as a SPECT tracer for CXCR4 receptor imaging J. Ranalytic Nucl Chem. (2021) 327:627–33. doi: 10.1007/s10967-020-07532-8

[77] von Spreckelsen N Fadzen CM Hartrampf N Ghotmi Y Wolfe JM Dubey S. Targeting glioblastoma using a novel peptide specific to a deglycosylated isoform of brevican. Adv Therapeutic. (2021) 4:2000244. doi: 10.1002/adtp.202000244, PMID: 33997269 PMC8114962

[78] Cruz-Nova P Ocampo-García B Carrión-Estrada DA Briseño-Diaz P Ferro-Flores G Jiménez-Mancilla N. 131I-C19 iodide radioisotope and synthetic I-C19 compounds as K-Ras4B-PDE6δ Inhibitors: A novel approach against colorectal cancer-biological characterization, biokinetics and dosimetry. Molecules. (2022) 27:5446. doi: 10.3390/molecules27175446, PMID: 36080216 PMC9458062

[79] Su PL Chakravarthy K Furuya N Brownstein J Yu JH Long MX. DLL3-guided therapies in small-cell lung cancer: from antibody-drug conjugate to precision immunotherapy and radio-immunotherapy. Mol Cancer. (2024) 23:97. doi: 10.1186/s12943-024-02012-z, PMID: 38730427 PMC11084107

[80] Guan SS Wu CT Liao TZ Lin KL Peng CL Shih YH. A novel 111indium-labeled dual carbonic anhydrase 9-targeted probe as a potential SPECT imaging radiotracer for detection of hypoxic colorectal cancer cells. Eur J Pharmaceut Biopharmaceut. (2021) 168:38–52. doi: 10.1016/j.ejpb.2021.08.004, PMID: 34450241

[81] de Campos NSP Souza BS da Silva GCP Porto VA Chalbatani GM Lagreca G. Carbonic anhydrase IX: A renewed target for cancer immunotherapy. Cancers. (2022) 14:1392. doi: 10.3390/cancers14061392, PMID: 35326544 PMC8946730

[82] Li X Li Z Huang MX Wang R Li MF Yang H. Gallium-68-labeled Z PDGFRβ Affibody: A potential PET probe for platelet-derived growth factor receptor β-expressing carcinomas. Mol Pharmaceut. (2023) 20:1357–64. doi: 10.1021/acs.molpharmaceut.2c00957, PMID: 36692381

[83] Liu WH Tang Y Ma H Li FZ Hu YJ Yang YY. Astatine-211 labelled a small molecule peptide: specific cell killing *in vitro* and targeted therapy in a nude-mouse model. Radiochinica Acta. (2021) 109:119–26. doi: 10.1515/ract-2020-0016

[84] Thakur ML Tripathi SK Gomella LG Salmanoglu E Kim S Kelly WK. Imaging urothelial bladder cancer: A VPAC PET targeted approach. Can J Urol. (2021) 28:10596–602., PMID: 33872557

[85] Takashima H Koga Y Manabe S Ohnuki K Tsumura R Anzai T. Radioimmunotherapy with an 211At-labeled anti-tissue factor antibody protected by sodium ascorbate. Cancer Sci. (2021) 112:1975–86. doi: 10.1111/cas.14857, PMID: 33606344 PMC8088967

[86] Laszlo GS Orozco JJ Kehret AR Lunn MC Huo J Hamlin DK. Development of [211At]astatine-based anti-CD123 radioimmunotherapy for acute leukemias and other CD123+malignancies. Leukemia. (2022) 36:1485–91. doi: 10.1038/s41375-022-01580-7, PMID: 35474099 PMC9177726

[87] Trencsényi G Halmos G Képes Z. Radiolabeled NGR-based heterodimers for angiogenesis imaging: A review of preclinical studies. Cancers. (2023) 15:4459. doi: 10.3390/cancers15184459, PMID: 37760428 PMC10526435

[88] Yang YC Wang J Liu W Deng H Zhao P Liao W. 89Zr and 177Lu labeling of anti-DR5 monoclonal antibody for colorectal cancer targeting PET-imaging and radiotherapy. J Radioanalytical Nucl Chem. (2021) 330:997–1005. doi: 10.1007/s10967-021-07979-3

[89] Cuda TJ He YW Kryza T Khan T Tse BW Sokolowski KA. Preclinical molecular PET-CT imaging targeting CDCP1 in colorectal cancer. Contrast Media Mol Imag. (2021), 3153278. doi: 10.1155/2021/3153278, PMID: 34621145 PMC8455202

[90] Montemagno C Raes F Ahmadi M Bacot S Debiossat M Leenhardt J. *In vivo* biodistribution and efficacy evaluation of NeoB, A radiotracer targeted to GRPR, in mice bearing gastrointestinal stromal tumor. Cancers. (2021) 13:1051. doi: 10.3390/cancers13051051, PMID: 33801382 PMC7958597

[91] Anderson NM Simon MC. The tumor microenvironment. Curr Biol. (2020) 30:R921–5. doi: 10.1016/j.cub.2020.06.081, PMID: 32810447 PMC8194051

[92] Yang D Liu J Qian H Zhuang Q. Cancer-associated fibroblasts: from basic science to anticancer therapy. Exp Mol Med. (2023) 55:1322–32. doi: 10.1038/s12276-023-01013-0, PMID: 37394578 PMC10394065

[93] Oster C Kessler L Blau T Keyvani K Pabst KM Fendler WP. The role of fibroblast activation protein in glioblastoma and gliosarcoma: A comparison of tissue, 68Ga-FAPI-46 PET data, and survival data. J Nucl Med. (2024) 65:1217–23. doi: 10.2967/jnumed.123.267151, PMID: 38960714

[94] Unterrainer LM Eismann L Lindner S Gildehaus FJ Toms J Casuscelli J. 68 Ga]Ga-FAPI-46 PET/CT for locoregional lymph node staging in urothelial carcinoma of the bladder prior to cystectomy: initial experiences from a pilot analysis. Eur J Nucl Med Mol Imaging. (2024) 51:1786–9. doi: 10.1007/s00259-024-06595-z, PMID: 38236427 PMC11043110

[95] Wegen S Claus K Linde P Rosenbrock J Trommer M Zander T. Impact of FAPI-46/dual-tracer PET/CT imaging on radiotherapeutic management in esophageal cancer. Radiat Oncol. (2024) 19:44. doi: 10.1186/s13014-024-02430-9, PMID: 38575990 PMC10993448

[96] Metzger G Bayerl C Rogasch JM Furth C Wetz C Beck M. 68Ga-labeled fibroblast activation protein inhibitor (FAPI) PET/CT for locally advanced or recurrent pancreatic cancer staging and restaging after chemoradiotherapy. Theranostics. (2024) 14:4184–97. doi: 10.7150/thno.95329, PMID: 39113796 PMC11303068

[97] Chen L Zheng S Chen L Xu S Wu K Kong L. 68Ga-labeled fibroblast activation protein inhibitor PET/CT for the early and late prediction of pathologic response to neoadjuvant chemotherapy in breast cancer patients: A prospective study. J Nucl Med. (2023) 64:1899–905. doi: 10.2967/jnumed.123.266079, PMID: 37918866 PMC10690122

[98] Has Simsek D Guzel Y Denizmen D Sanli Y Buyukkaya F Kovan B. The inferior performance of [68Ga]Ga-FAPI-04 PET/CT as a diagnostic and theragnostic biomarker in [177Lu]Lu-DOTATATE refractory well-differentiated neuroendocrine tumors. Eur J Nucl Med Mol Imaging. (2024) 51:828–40. doi: 10.1007/s00259-023-06497-6, PMID: 37947850

[99] Pabst KM Mei R Lückerath K Hadaschik BA Kesch C Rawitzer J. Detection of tumour heterogeneity in patients with advanced, metastatic castration-resistant prostate cancer on [68Ga]Ga-/[18F]F-PSMA-11/-1007, [68Ga]Ga-FAPI-46 and 2-[18F]FDG PET/CT: a pilot study. Eur J Nucl Med Mol Imaging. (2024) 52:342–53. doi: 10.1007/s00259-024-06891-8, PMID: 39207485 PMC11599349

[100] Kuyumcu S Kovan B Sanli Y Buyukkaya F Simsek DH Özkan ZG. Safety of fibroblast activation protein-targeted radionuclide therapy by a low-dose dosimetric approach using 177Lu-FAPI04. Clin Nucl Med. (2021) 46:641–6. doi: 10.1097/RLU.0000000000003667, PMID: 33883494

[101] Loktev A Lindner T Burger EM Altmann A Giesel F Kratochwil C. Development of fibroblast activation protein-targeted radiotracers with improved tumor retention. J Nucl Med. (2019) 60:1421–9. doi: 10.2967/jnumed.118.224469, PMID: 30850501 PMC6785792

[102] Ferdinandus J Costa PF Kessler L Weber M Hirmas N Kostbade K. Initial clinical experience with 90Y-FAPI-46 radioligand therapy for advanced-stage solid tumors: A case series of 9 patients. J Nucl Med. (2022) 63:727–34. doi: 10.2967/jnumed.121.262468, PMID: 34385340 PMC9051597

[103] Fendler WP Pabst KM Kessler L Fragoso Costa P Ferdinandus J Weber M. Safety and efficacy of 90Y-FAPI-46 radioligand therapy in patients with advanced sarcoma and other cancer entities. Clin Cancer Res. (2022) 28:4346–53. doi: 10.1158/1078-0432.CCR-22-1432, PMID: 35833949 PMC9527500

[104] Hamacher R Pabst KM Cheung PF Heilig CE Hüllein J Liffers ST. Fibroblast activation protein α-directed imaging and therapy of solitary fibrous tumor. J Nucl Med. (2024) 65:252–7. doi: 10.2967/jnumed.123.266411, PMID: 38176718

[105] Assadi M Rekabpour SJ Jafari E Divband G Nikkholgh B Amini H. Feasibility and therapeutic potential of 177Lu-fibroblast activation protein inhibitor-46 for patients with relapsed or refractory cancers: A preliminary study. Clin Nucl Med. (2021) 46:e523–30. doi: 10.1097/RLU.0000000000003810, PMID: 34269729

[106] Zhong X Guo JR Han XP Yang R Zhang J Shao GQ. Synthesis and preclinical evaluation of a novel FAPI-04 dimer for cancer theragnostics. Mol Pharmaceut. (2023) 20:2402–14. doi: 10.1021/acs.molpharmaceut.2c00965, PMID: 37015025

[107] Pang Y Zhao L Fang J Chen J Meng L Sun L. Development of FAPI tetramers to improve tumor uptake and efficacy of FAPI radioligand therapy. J Nucl Med. (2023) 64:1449–55. doi: 10.2967/jnumed.123.265599, PMID: 37321827 PMC10478824

[108] Ballal S Yadav MP Moon ES Kramer VS Roesch F Kumari S. First-in-human results on the biodistribution, pharmacokinetics, and dosimetry of [177Lu]Lu-DOTA.SA.FAPi and [177Lu]Lu-DOTAGA.(SA.FAPi)2. Pharm (Basel). (2021) 14:1212. doi: 10.3390/ph14121212, PMID: 34959613 PMC8707268

[109] Banihashemian SS Divband G Pirayesh E Nikkholgh B Amini H Shahrnoy AA. 68Ga -FAP-2286, a novel promising theragnostic approach for PET/CT imaging in patients with various type of metastatic cancers. Eur J Nucl Med Mol Imag. (2024) 51:1981–8. doi: 10.1007/s00259-024-06635-8, PMID: 38376804

[110] Baum RP Schuchardt C Singh A Chantadisai M Robiller FC Zhang JJ. Feasibility, biodistribution, and preliminary dosimetry in peptide-targeted radionuclide therapy of diverse adenocarcinomas using 177Lu-FAP-2286: first-in-humans results. J Nucl Med. (2022) 63:415–23. doi: 10.2967/jnumed.120.259192, PMID: 34168013 PMC8978187

[111] Cui XY Li Z Kong ZR Liu Y Meng H Wen ZH. Covalent targeted radioligands potentiate radionuclide therapy. Nat. (2024) 630:206–13. doi: 10.1038/s41586-024-07461-6, PMID: 38778111

[112] Xu JF Li SH Xu SS Dai J Luo ZG Cui JJ. Screening and preclinical evaluation of novel radiolabeled anti-fibroblast activation protein-α Recombinant antibodies. Cancer Biother Radiopharmaceut. (2022) 38:726–37. doi: 10.1089/cbr.2021.0389, PMID: 35612467

[113] Xu MX Chen JY Zhang P Cai J Song HB Li Z Liu ZB. An antibody-radionuclide conjugate targets fibroblast activation protein for cancer therapy. Eur J Nucl Med Mol Imag. (2023) 50:3214–24. doi: 10.1007/s00259-023-06300-6, PMID: 37318538

[114] Liu Y Tang HC Song TC Xu MX JY C Cui XY. Organotrifluoroborate enhances tumor targeting of fibroblast activation protein inhibitors for targeted radionuclide therapy. Eur J Nucl Med Mol Imag. (2023) 50:2636–46. doi: 10.1007/s00259-023-06230-3, PMID: 37103565

[115] Aso A Kaneda-Nakashima K Nabetani H Kadonaga Y Shirakami Y Watabe T. Substrate study for dihydroxyboryl astatine substitution reaction with fibroblast activation protein inhibitor (FAPI). Chem Lett. (2022) 51:1091–4. doi: 10.1246/cl.220391

[116] Chu Y Awasthi A Lee S Edani D Yin C Hochberg J. Obinutuzumab (GA101) vs. rituximab significantly enhances cell death, antibody-dependent cytotoxicity and improves overall survival against CD20+ primary mediastinal B-cell lymphoma (PMBL) in a xenograft NOD-scid IL2Rgnull (NSG) mouse model: a potential targeted agent in the treatment of PMBL. Oncotarget. (2020) 11:3035–47. doi: 10.18632/oncotarget.27691, PMID: 32850008 PMC7429176

[117] Kimball AS Webb TJ. The roles of radiotherapy and immunotherapy for the treatment of lymphoma. Mol Cell Pharmacol. (2013) 5:27–38., PMID: 24648864 PMC3955882

[118] Grillo-López AJ. Zevalin: the first radioimmunotherapy approved for the treatment of lymphoma. Expert Rev Anticancer Ther. (2002) 2:485–93. 10.1586/14737140.2.5.485 12382517

[119] Durando M Gopal AK Tuscano J Persky D. A systematic review of clinical applications of anti-CD20 radioimmunotherapy for lymphoma. Oncologist. (2024) 29:278–88. doi: 10.1093/oncolo/oyad333, PMID: 38207010 PMC10994254

[120] Rogoza O Megnis K Kudrjavceva M Gerina-Berzina A Rovite V. Role of somatostatin signalling in neuroendocrine tumours. Int J Mol Sci. (2022) 23:1447. doi: 10.3390/ijms23031447, PMID: 35163374 PMC8836266

[121] Triebel J Robles JP Zamora M Clapp C Bertsch T. New horizons in specific hormone proteolysis. Trends Endocrinol Metab. (2022) 33:371–7. doi: 10.1016/j.tem.2022.03.004, PMID: 35397984

[122] Ambrosini V Fani M Fanti S Forrer F Maecke HR. Radiopeptide imaging and therapy in Europe. Eur J Nucl Med. (2011) 52:42–54. doi: 10.2967/jnumed.110.085753, PMID: 22144555

[123] Carideo L Prosperi D Panzuto F Magi L Pratesi MS Rinzivillo M. Role of combined [68Ga] Ga-DOTA-SST analogues and [18F]FDG PET/CT in the management of GEP-NENs: A systematic review. J Clin Med. (2019) 8:1032. doi: 10.3390/jcm8071032, PMID: 31337043 PMC6678236

[124] Strosberg J El-Haddad G Wolin E Hendifar A Yao J Chasen B. Phase3 trial of (177)Lu-dotatate for midgut neuroendocrine tumors. N Engl J Med. (2017) 376:125–35. doi: 10.1056/NEJMoa1607427, PMID: 28076709 PMC5895095

[125] Ballal S Yadav MP Bal C Sahoo RK Tripathi M. Broadening horizons with 225Ac-DOTATATE targeted alpha therapy for gastroenteropancreatic neuroendocrine tumour patients stable or refractory to 177Lu-DOTATATE PRRT: first clinical experience on the efficacy and safety. Eur J Nucl Med Mol Imaging. (2020) 47:934–46. doi: 10.1007/s00259-019-04567-2, PMID: 31707430

[126] Conlon KC Sportes C Brechbiel MW Fowler DH Gress R Miljkovic MD. 90Y-daclizumab (Anti-CD25), high-dose carmustine, etoposide, cytarabine, and melphalan chemotherapy and autologous hematopoietic stem cell transplant yielded sustained complete remissions in 4 patients with recurrent Hodgkin’s lymphoma. Cancer Biother Radiopharm. (2020) 35:249–61. doi: 10.1089/cbr.2019.3298, PMID: 32275165 PMC7247027

[127] Cremonesi M Ferrari ME Bodei L Chiesa C Sarnelli A Garibaldi C. Correlation of dose with toxicity and tumour response to 90Y- and 177Lu-PRRT provides the basis for optimization through individualized treatment planning. Eur J Nucl Med Mol Imaging. (2018) 45:2426–41. doi: 10.1007/s00259-018-4044-x, PMID: 29785514 PMC6716520

[128] Baum RP Zhang J Schuchardt C Müller D Mäcke H. First-in humans study of the SSTR antagonist 177Lu-DOTA-LM3 for peptide receptor radionuclide therapy in patients with metastatic neuroendocrine neoplasms: dosimetry, safety, and efficacy. J Nucl Med. (2021) 62:1571–81. doi: 10.2967/jnumed.120.258889, PMID: 33674401 PMC8612334

[129] Dalm SU Haeck J Doeswijk GN de Blois E de Jong M van Deurzen CHM. SSTR-mediated imaging in breast cancer: is there a role for radiolabeled somatostatin receptor antagonists? J Nucl Med. (2017) 58:1609–14. doi: 10.2967/jnumed.116.189035, PMID: 28450563

[130] Rubira L Deshayes E Santoro L Kotzki PO Fersing C. 225Ac-labeled somatostatin analogs in the management of neuroendocrine tumors: from radiochemistry to clinic. Pharmaceutics. (2023) 15:1051. doi: 10.3390/pharmaceutics15041051, PMID: 37111537 PMC10146019

[131] Li F Yu Y Jiang M Zhang H. Targets for improving prostate tumor response to radiotherapy. Eur J Pharmacol. (2025) 986:177149. doi: 10.1016/j.ejphar.2024.177149, PMID: 39577551

[132] Oldan JD Almaguel F Voter AF Duran A Gafita A Pomper MG. PSMA-targeted radiopharmaceuticals for prostate cancer diagnosis and therapy. Cancer J. (2024) 30:176–84. doi: 10.1097/PPO.0000000000000718, PMID: 38753752

[133] Eiber M Fendler WP Rowe SP Calais J Hofman MS Maurer T. Prostate-specific membrane antigen ligands for imaging and therapy. J Nucl Med. (2017) 58:67S–76S. doi: 10.2967/jnumed.116.1867674 28864615

[134] Groener D Baumgarten J Haefele S Happel C Klimek K Mader N. Salvage radioligand therapy with repeated cycles of 177Lu-PSMA-617 in metastatic castration-resistant prostate cancer with diffuse bone marrow involvement. Cancers. (2021) 13:4017. doi: 10.3390/cancers13164017, PMID: 34439172 PMC8393804

[135] Giunta EF Brighi N Gurioli G Matteucci F Paganelli G De Giorgi U. 177Lu-PSMA therapy in metastatic prostate cancer: An updated review of prognostic and predictive biomarkers. Cancer Treat Rev. (2024) 125:102699. doi: 10.1016/j.ctrv.2024.102699, PMID: 38422894

[136] Stangl-Kremser J Ricaurte-Fajardo A Subramanian K Osborne JR Sun MC Tagaw ST. Response to RL-225Ac in prostate cancer: Effect of prior treatment with RL-177Lu: A systematic review of the literature. Prostate. (2023) 83:901–11. doi: 10.1002/pros.24531, PMID: 36960580

[137] Muniz M Loprinzi CL Orme JJ Koch RM Mahmoud AM Kase AM. Salivary toxicity from PSMA-targeted radiopharmaceuticals: What we have learned and where we are going. Cancer Treat Rev. (2024) 127:102748. doi: 10.1016/j.ctrv.2024.102748, PMID: 38703593 PMC11160931

[138] Tagawa ST Sun M Sartor AO Thomas C Singh S Bissassar M. Phase I study of 225Ac-J591 for men with metastatic castration-resistant prostate cancer (mCRPC). J Clin Oncol. (2021) 39:5015. doi: 10.1200/JCO.2021.39.15_suppl.5015 PMC10906595 37922438

[139] Picciotto M FranChina T Russo A Ricciardi GRR Provazza G Sava S. Emerging role of Radium-223 in the growing therapeutic armamentarium of metastatic castration-resistant prostate cancer. Expert Opin Pharmacother. (2017) 18:899–908. doi: 10.1080/14656566.2017.1323875, PMID: 28449621

[140] Higano CS George DJ Shore ND Sartor O Miller K Conti PS. Clinical outcomes and treatment patterns in REASSURE: planned interim analysis of a real-world observational study of radium-223 in metastatic castration-resistant prostate cancer. EClinicalMedicine. (2023) 60:101993. doi: 10.1016/j.eclinm.2023.101993, PMID: 37251627 PMC10209672

[141] Ling SW van der Veldt AAM Konijnenberg M Segbers M Hooijman E Bruchertseifer F. Evaluation of the tolerability and safety of 225Ac -PSMA-I&T in patients with metastatic prostate cancer: a phase I dose escalation study. BMC Cancer. (2024) 24:146. doi: 10.1186/s12885-024-11900-y, PMID: 38287346 PMC10826262

[142] Hagemann UB Wickstroem K Hammer S Bjerke RM Zitzmann-Kolbe S Ryan OB. Advances in precision oncology: targeted thorium-227 conjugates as a new modality in targeted alpha therapy. Cancer Biother Radiopharm. (2020) 35:497–510. doi: 10.1089/cbr.2020.3568, PMID: 32255671 PMC7475103

[143] Kozempel J Mokhodoeva O Vlk M. Progress in targeted alpha-particle therapy. What we learned about recoils release from *in vivo* generators. Molecules. (2018) 23:581. doi: 10.3390/molecules23030581, PMID: 29510568 PMC6017877

[144] Ahenkorah S Cassells I Deroose CM Cardinaels T Burgoyne AR Bormans G. Bismuth-213 for targeted radionuclide therapy: from atom to bedside. Pharmaceutics. (2021) 13:599. doi: 10.3390/pharmaceutics13050599, PMID: 33919391 PMC8143329

[145] Muslimov AR Antuganov D Tarakanchikova YV Karpov TE Zhukov MV Zyuzin MV. An investigation of calcium carbonate core-shell particles for incorporation of 225Ac and sequester of daughter radionuclides: *In vitro* and *in vivo* studies. J Control Release. (2021) 330:726–37. doi: 10.1016/j.jconrel.2021.01.008, PMID: 33428985

[146] Mdanda S Ngema LM Mdlophane A Sathekge MM Zeevaart JR. Recent innovations and nano-delivery of actinium-225: A narrative review. Pharmaceutics. (2023) 15:1719. doi: 10.3390/pharmaceutics15061719, PMID: 37376167 PMC10304099

[147] Toro-González M Akingbesote N Bible A Pal D Sanders B Ivanov AS. Development of 225Ac-doped biocompatible nanoparticles for targeted alpha therapy. J Nanobiotech. (2024) 22:306. doi: 10.1186/s12951-024-02520-6, PMID: 38825717 PMC11145892

[148] Karpov TE Muslimov AR Antuganov DO Postovalova AS Pavlov DA. Usov, Impact of metallic coating on the retention of 225Ac and its daughters within core-shell nanocarriers. J Colloid Interface Sci. (2021) 608:2571–83. doi: 10.1016/j.jcis.2021.10.187, PMID: 34801240

[149] Feng YT Meshaw RL Finch SW Zheng YX Minn I Vaidyanathan G. A third generation PSMA-targeted agent 211At: Synthesis and *in vivo* evaluation. Nucl Med Biol. (2024) 134:108916. doi: 10.1016/j.nucmedbio.2024.108916, PMID: 38703587 PMC11180594

[150] Bauer D Carter LM Atmane MI De Gregorio R Michel A Kaminsky S. 212Pb-pretargeted theragnostics for pancreatic cancer. J Nucl Med. (2024) 65:109–16. doi: 10.2967/jnumed.123.266388, PMID: 37945380 PMC10755526

[151] Pallares RM Abergel RJ. Development of radiopharmaceuticals for targeted alpha therapy: Where do we stand? Front Med. (2022) 9:1020188. doi: 10.3389/fmed.2022.1020188, PMID: 36619636 PMC9812962

[152] Trujillo-Nolasco M Morales-Avila E Cruz-Nova P Katti KV Ocampo-García B. Nanoradiopharmaceuticals based on alpha emitters: recent developments for medical applications. Pharmaceutics. (2021) 13:1123. doi: 10.3390/pharmaceutics13081123, PMID: 34452084 PMC8398190

[153] Najdian A Beiki D Abbasi M Gholamrezanezhad A Ahmadzadehfar H Amani M. Exploring innovative strides in radiolabeled nanoparticle progress for multimodality cancer imaging and theranostic applications. Cancer Imaging. (2024) 24:127. doi: 10.1186/s40644-024-00762-z, PMID: 39304961 PMC11416024

[154] Murray I Rojas B Gear J Callister R Cleton A Flux GD. Quantitative dual-isotope planar imaging of thorium-227 and radium-223 using defined energy windows. Cancer Biother Radiopharmaceuticals. (2020) 35:530–9. doi: 10.1089/cbr.2019.3554, PMID: 32429699 PMC7475104

[155] Ma J Li L Liao T Gong W Zhang C. Efficacy and safety of 225Ac-PSMA-617-targeted alpha therapy in metastatic castration-resistant prostate cancer: A systematic review and meta-analysis. Front Oncol. (2022) 12:796657. doi: 10.3389/fonc.2022.796657, PMID: 35186737 PMC8852230

[156] Kratochwil C Schmidt K Afshar-Oromieh A Bruchertseifer F Rathke H Morgenstern A. Targeted alpha therapy of mCRPC: Dosimetry estimate of 213Bismuth-PSMA-617. Eur J Nucl Med Mol Imaging. (2018) 45:31–7. doi: 10.1007/s00259-017-3817-y, PMID: 28891033 PMC5700223

[157] Meredith RF Buchsbaum DJ. Pretargeted radioimmunotherapy. Int J Radiat Oncol Biol Phys. (2006) 66:S57–9. doi: 10.1016/j.ijrobp.2006.04.058, PMID: 16979441

[158] Timperanza C Jensen H Hansson E Bäck T Lindegren S Aneheim E. *In vitro* and *in vivo* evaluation of a tetrazine-conjugated poly-L-lysine effector molecule labeled with astatine-211. EJNMMI Radiopharm Chem. (2024) 9:43. doi: 10.1186/s41181-024-00273-z, PMID: 38775973 PMC11111624

[159] Timperanza C Jensen H Bäck T Lindegren S Aneheim E. Pretargeted alpha therapy of disseminated cancer combining click chemistry and astatine-211. Pharmaceutics. (2023) 16:595. doi: 10.3390/ph16040595, PMID: 37111352 PMC10145095

[160] Keinänen O Fung K Brennan JM Zia N Harris M van Dam E. Harnessing 64Cu/67Cu for a theragnostic approach to pretargeted radioimmunotherapy. Proc Natl Acad Sci. (2020) 117:28316–27. doi: 10.1073/pnas.2009960117, PMID: 33106429 PMC7668034

[161] Santich BH Cheal SM Ahmed M McDevitt MR Ouerfelli O Yang GB. A self-assembling and disassembling (SADA) bispecific antibody (BsAb) platform for curative two-step pretargeted radioimmunotherapy. Clin Cancer Res. (2021) 27:532–41. doi: 10.1158/1078-0432.CCR-20-2150, PMID: 32958698 PMC7855367

[162] Sartor O de Bono J Chi KN Fizazi K Herrmann K Rahbar K. VISION investigators. Lutetium-177-PSMA-617 for metastatic castration-resistant prostate cancer. N Engl J Med. (2021) 385:1091–103. doi: 10.1056/NEJMoa2107322, PMID: 34161051 PMC8446332

[163] Yang MD Liu HP Lou JJ Zhang JJ Zuo CJ Zhu MQ. Alpha-emitter radium-223 induces STING-dependent pyroptosis to trigger robust antitumor immunity. Small. (2024) 20:2307448. doi: 10.1002/smll.202307448, PMID: 37845027

[164] Yi M Jiao D Xu H Liu Q Zhao W Han X. Biomarkers for predicting efficacy of PD-1/PD-L1 inhibitors. Mol Cancer. (2018) 17:129. doi: 10.1186/s12943-018-0864-3, PMID: 30139382 PMC6107958

[165] Lunj S Smith TAD Reeves KJ Currell F Honeychurch J Hoskin P. Immune effects of α and β radionuclides in metastatic prostate cancer. Nat Rev Urol. (2024) 21:651–61. doi: 10.1038/s41585-024-00924-5, PMID: 39192074

[166] Zboralski D Osterkamp F Christensen E Bredenbeck A Schumann A Hoehne A. Fibroblast activation protein targeted radiotherapy induces an immunogenic tumor microenvironment and enhances the efficacy of PD-1 immune checkpoint inhibition. Eur J Nucl Med Mol Imag. (2023) 50:2621–35. doi: 10.1007/s00259-023-06211-6, PMID: 37086273 PMC10317891

[167] Hasanov E Flynt L Slack Tidwell R Hwang H Brooks R Wood LM. STARLITE 1: Phase 1b/2 study of combination 177Lu girentuximab plus cabozantinib and nivolumab in treatment naïve patients with advanced clear cell RCC. Oncologist. (2023) 28:S13. doi: 10.1093/oncolo/oyad216.021, PMID: 40288950

[168] Malo ME Allen KJH Jiao RB Frank C Rickles D Dadachova E. Mechanistic insights into synergy between melanin-targeting radioimmunotherapy and immunotherapy in experimental melanoma. Int J Mol Sci. (2020) 21:8721. doi: 10.3390/ijms21228721, PMID: 33218169 PMC7698872

[169] Sheykhhasan M Ahmadieh-Yazdi A Vicidomini R Poondla N Tanzadehpanah H Dirbaziyan A. CAR T therapies in multiple myeloma: unleashing the future. Cancer Gene Ther. (2024) 31:667–86. doi: 10.1038/s41417-024-00750-2, PMID: 38438559 PMC11101341

[170] Sodji QH Forsberg MH Cappabianca D Kerr CP Sarko L Shea A. Comparative study of the effect of radiation delivered by lutetium-177 or actinium-225 on anti-GD2 chimeric antigen receptor T cell viability and functions. Cancers. (2024) 16:191. doi: 10.3390/cancers16010191, PMID: 38201618 PMC10778389

[171] Hall E Hussain SA Porta N Lewis R Crundwell M Jenkins P. Chemoradiotherapy in muscle-invasive bladder cancer: 10- yr follow-up of the phase 3 randomised controlled BC2001 trial. Eur Urol. (2022) 82:273–9. doi: 10.1016/j.eururo.2022.04.017, PMID: 35577644

[172] Gilmer TM Lai CH Guo KX Deland K Ashcraft KA Stewart AE. A novel dual ATM/DNA-PK inhibitor, XRD-0394, potently radiosensitizes and potentiates PARP and topoisomerase I inhibitors. Mol Cancer Therapeut. (2024) 23:751–65. doi: 10.1158/1535-7163.MCT-23-0890, PMID: 38588408

[173] di Santo G Santo G Sviridenko A Virgolini I. Peptide receptor radionuclide therapy combinations for neuroendocrine tumours in ongoing clinical trials: status 2023. Theragnostics. (2024) 14:940–53. doi: 10.7150/thno.91268, PMID: 38250038 PMC10797289

[174] Pavlakis N Randsom D Wyld D Sjoquist K Wilson K Gebski V. Australasian Gastrointestinal Trials Group (AGITG) CONTROL NET Study: 177Lu-DOTATATE peptide receptor radionuclide therapy (PRRT) and capecitabine plus temozolomide (CAPTEM) for pancreas and midgut neuroendocrine tumours (pNETS, mNETS)—Final results. J Clin Oncol. (2022) 40:4122–2. doi: 10.1200/JCO.2022.40.16_suppl.4122

[175] Jeggo P Lavin MF. Cellular radiosensitivity: how much better do we understand it? Int J Radiat Biol. (2009) 85:1061–81. doi: 10.3109/09553000903261263, PMID: 19995233

[176] Kim D Nam HJ. PARP inhibitors: clinical limitations and recent attempts to overcome them. Int J Mol Sci. (2022) 23:8412. doi: 10.3390/ijms23158412, PMID: 35955544 PMC9369301

[177] Anscher MS Chang E Gao X Gong Y Weinstock C Bloomquist E. FDA approval summary: rucaparib for the treatment of patients with deleterious BRCA-mutated metastatic castrate-resistant prostate cancer. Oncologist. (2021) 26:139–46. doi: 10.1002/onco.13585, PMID: 33145877 PMC7873319

[178] Bao GF Zhou HM Zou SJ Chen LX Zhang BC Wang ZQ. Inhibition of poly(ADP-ribose) polymerase sensitizes [Lu]Lu-DOTAGA.(SA.FAPi) mediated radiotherapy in triple-negative breast cancer. Mol Pharmaceut. (2023) 20:2443–51. doi: 10.1021/acs.molpharmaceut.2c01051, PMID: 37067162

[179] Qin Y Imobersteg S Frank S Blanc A Chiorazzo T Berger P. Signaling network response to a-particle-targeted therapy with the 225Ac-labeled minigastrin analog 225Ac-PP-F11N reveals the radiosensitizing potential of histone deacetylase inhibitors. J Nucl Med. (2023) 64:873–9. doi: 10.2967/jnumed.122.264597, PMID: 36732057 PMC10241010

[180] Peeken JC Vaupel P Combs SE. Integrating hyperthermia into modern radiation oncology: what evidence is necessary? Front Oncol. (2017) 7:132. doi: 10.3389/fonc.2017.00132, PMID: 28713771 PMC5492395

[181] Simón M Jorgensen JT Khare HA Christensen C Nielsen CH Kjaer A. Combination of [177Lu]Lu-DOTA-TATE targeted radionuclide therapy and photothermal therapy as a promising approach for cancer treatment: *in vivo* studies in a human xenograft mouse model. Pharmaceuticals. (2022) 14:1284. doi: 10.3390/pharmaceutics14061284, PMID: 35745856 PMC9227845

[182] Zhang C Liu J Wu J Ranjan K Cui X Wang X. Key molecular DNA damage responses of human cells to radiation. Front Cell Dev Biol. (2024) 12:1422520. doi: 10.3389/fcell.2024.1422520, PMID: 39050891 PMC11266142

[183] An J Tang S Hong G Chen W Chen M Song J. An unexpected strategy to alleviate hypoxia limitation of photodynamic therapy by biotinylation of photosensitizers. Nat Commun. (2022) 13:2225. doi: 10.1038/s41467-022-29862-9, PMID: 35469028 PMC9038921

[184] Jo C Ahn H Kim JH Lee YJ Kim JY Lee KC. Cancer therapy by antibody-targeted Cerenkov light and metabolism-selective photosensitization. J Controlled Res. (2022) 352:25–34. doi: 10.1016/j.jconrel.2022.10.014, PMID: 36243234

[185] Jiang YF Wang AQ Wu QH Jia GP Hai WX Zhang M. 89Zr-labeled pH-responsive gold nanoclusters for radiosensitization therapy of tumors. ACS Appl Nano Mater. (2024) 7:15325–33. doi: 10.1021/acsanm, PMID: 39744149

[186] Gong L Zhang Y Liu C Zhang M Han S. Application of radiosensitizers in cancer radiotherapy. Int J Nanomed. (2021) 16:1083–102. doi: 10.2147/IJN.S290438, PMID: 33603370 PMC7886779

[187] Smith TAD West CML Joseph N Lane B Irlam-Jones J More E. A hypoxia biomarker does not predict benefit from giving chemotherapy with radiotherapy in the BC2001 randomised controlled trial. EBioMedicine. (2024) 101:105032. doi: 10.1016/j.ebiom.2024.105032, PMID: 38387404 PMC10897900

[188] Koll FJ Döring C Herwig L Hoeh B Wenzel M Cano Garcia C. Impact of consensus molecular subtypes on survival with and without adjuvant chemotherapy in muscle-invasive urothelial bladder cancer. J Clin Pathol. (2023) 78:19–27. doi: 10.1136/jcp-2023-208973. jcp-2023-208973., PMID: 37989554

[189] Scott JG Berglund A Schell MJ Mihaylov I Fulp WJ Yue B. A genome-based model for adjusting radiotherapy dose (GARD): a retrospective, cohort-based study. Lancet Oncol. (2017) 18:202–11. doi: 10.1016/S1470-2045(16)30648-9, PMID: 27993569 PMC7771305

[190] Smith TAD. Gene abnormalities and modulated gene expression associated with radionuclide treatment: towards predictive biomarkers of response. Genes (Basel). (2024) 15:688. doi: 10.3390/genes15060688, PMID: 38927624 PMC11202453

[191] Alsaed B Lin L Son J Li J Smolander J Lopez T. Intratumor heterogeneity of EGFR expression mediates targeted therapy resistance and formation of drug tolerant microenvironment. Nat Commun. (2025) 16:28. doi: 10.1038/s41467-024-55378-5, PMID: 39747003 PMC11695629

[192] Hoskin PJ Sibtain A Daley FM Wilson GD. GLUT1 and CAIX as intrinsic markers of hypoxia in bladder cancer: relationship with vascularity and proliferation as predictors of outcome of ARCON. Br J Cancer. (2003) 89:1290–7. doi: 10.1038/sj.bjc.6601260, PMID: 14520462 PMC2394309

